# Integrated analysis and validation of the TRIM28-H2AX-CDK4 diagnostic model assists to predict the progression of HCC

**DOI:** 10.18632/aging.205137

**Published:** 2023-10-20

**Authors:** Qifei Tian, Guofang Lu, Ying Ma, Lingling Ma, Yulong Shang, Ni Guo, Yan Huang, Lin Zhu, Rui Du

**Affiliations:** 1 Department of Gastroenterology, Dongying People’s Hospital, Dongying, Shandong 257091, China; 2 State Key Laboratory of Holistic Integrative Management of Gastrointestinal Cancers and National Clinical Research Center for Digestive Diseases, Xijing Hospital of Digestive Diseases, Fourth Military Medical University, Xi’an 710032, China; 3 Department of Physiology and Pathophysiology, National Key Discipline of Cell Biology, Fourth Military Medical University, Xi’an 710032, China; 4 Department of Gastroenterology, 941 Hospital of PLA, Xining, Qinghai 810007, China; 5 Department of Critical Medicine, 942 Hospital of PLA, Yin Chuan, Ning Xia, China; 6 Institute for Biomedical Sciences of Pain, Tangdu Hospital, Fourth Military Medical University, Xi’an 710038, China

**Keywords:** hepatocellular carcinoma, immune status, TRIM28, H2AX, CDK4

## Abstract

Hepatocellular carcinoma (HCC) is the second leading cause of cancer-related mortality in the world. However, identifying key genes that can be exploited for the effective diagnosis and management of HCC remains difficult. The study aims to examine the prognostic and diagnostic value of TRIM28-H2AX-CDK4 axis in HCC. Analysis in TCGA, GSEA and Gene expression profiling interactive analysis online tools were performed to explore the expression profiles of TRIM28, H2AX and CDK4. Data demonstrating the correlation between TRIM28 expression levels and immune infiltration states or the expression of genes associated with immune checkpoints genes were exacted from TCGA and TIMER. Genetic alteration and enrichment analysis were performed using the cBioPortal and GEPIA2 tools. Finally, the expression of these proteins in HCC was then examined and validated in an independent cohort using immunohistochemistry. TRIM28 alteration exhibited co-occurrence instead of mutual exclusivity with a large number of immune checkpoint components and tumor-infiltrating immune cells, especially B cells, were found to serve roles in patients with HCC with different TRIM28 expression levels. Higher expression levels of TRIM28, H2AX and CDK4 were associated with a poorer prognosis and recurrence in patients with HCC according to TCGA, which was validated further in an independent cohort of patients with HCC. Area under curve revealed the superior predictive power of applying this three-gene signatures in this validation cohort. The diagnostic model based on this TRIM28-H2AX-CDK4 signature is efficient and provides a novel strategy for the clinical management of HCC.

## INTRODUCTION

Hepatocellular carcinoma (HCC) is a type of liver cancer and is one of a number of cancer types that has demonstrated a sustained increase in incidence over the past decade. HCC is now the second leading cause of cancer-associated mortality in the world [1]. The poor prognosis and high mortality rates of HCC are partly due to the dysregulation of cell cycle progression [2] and the lack of sufficient tools for early diagnosis and effective surveillance [3]. It remains difficult to identify key genes that can be applied for the clinical management of patients with HCC.

Post-translational modifications (PTMs) take place in most, if not all, physiological processes and are critical mechanisms for regulating protein function. It has been previously reported that PTMs are associated with several processes of cancer, including proliferation, invasion and metastasis, drug resistance and suppression of apoptosis [4]. The ubiquitin-proteasome system is one type of a PTM system that has been reported to serve key roles not only in targeted protein degradation by the proteasome but also in the regulation of protein-protein interactions and enzyme activation. Ubiquitination serves an important role in the degradation of proteins that regulate cell cycle progression, intracellular signaling, DNA repair, protein quality control, transcriptional regulation and oncogenesis [5]. It has been documented that the hepatitis virus C core protein can upregulate E12/E47 expression levels by inhibiting their ubiquitin-dependent proteasomal degradation, which facilitated tumor invasion, migration and metastasis of hepatitis virus C-induced HCC [6]. Ubiquitination can regulate either tumor-suppressing and oncogenic pathways mainly by using ubiquitin ligases in cancer cells [7]. Therefore, it would be of great importance to screen for and validate ubiquitin ligases that can potentially serve key roles in the development of HCC. They can then be applied as candidate markers for the construction of novel tools to predict the prognosis of patients with HCC.

There are now >80 known tripartite motif (TRIM) variants in humans [5]. Accumulating evidence suggests that TRIM-containing proteins can regulate important intracellular processes, including intracellular signaling, cell cycle progression, innate immunity, transcription, autophagy, cell proliferation and oncogenesis [8–10]. Alterations in TRIM proteins have been reported to result in a variety of distinct pathological conditions, such as cardiovascular diseases, neuropsychiatric disorders, immunological diseases, musculoskeletal diseases, chromosomal abnormalities, developmental disorders and cancer [8, 5, 11]. Since the majority of proteins in the TRIM family contain a RING-finger domain, they are defined as E3 ubiquitin ligases [8]. It has been reported that TRIM serves important roles in the development of HCC. TRIM31 expression was found to be upregulated in HCC cell lines, which regulated the oncogenic mammalian target of rapamycin complex 1 pathway by promoting E3 ligase-mediated K48-linked ubiquitination and degradation [12]. In addition, upregulation of TRIM52 and TRIM28 expression was reported to promote HCC cell proliferation, migration and invasion [13, 14]. By contrast, TRIM16 can inhibit migration, invasion and epithelial-mesenchymal transition in HCC [15]. However, a systematic analysis of TRIM proteins should be performed to comprehensively characterize the role of TRIMs in HCC and to identify potential key TRIM genes in the HCC signaling hub from a holistic perspective.

In this study, data analysis in The Cancer Genome Atlas (TCGA), Gene Set Enrichment Analysis (GSEA) and Gene expression profiling interactive analysis, version 2 (GEPIA2) revealed that TRIM28 was the only TRIM gene that has exhibited increased expression levels in HCC and comprehensive association with the clinical outcomes of patients with HCC. We also evaluated the correlation of TRIM28 expression with various parameters of immune infiltration using Tumor Immune Estimation Resource (TIMER) database. Bioinformatics enrichment and univariate or multivariate Cox analysis found that phosphorylated histone H2A. X (H2AX) and cyclin-dependent kinase 4 (CDK4) are downstream functional targets of TRIM28. Combining TRIM28, H2AX and CDK4 expression with other clinical features, we also established a nomogram with capable of accurately predicting the outcomes of patients with HCC. Finally, we measured the expression levels and prognostic role of TRIM28, H2AX and CDK4 in an independent cohort of patients with HCC via IHC.

## RESULTS

### Upregulated expression of TRIM28 is predictive of a poor prognosis in HCC

**Figure 1:**
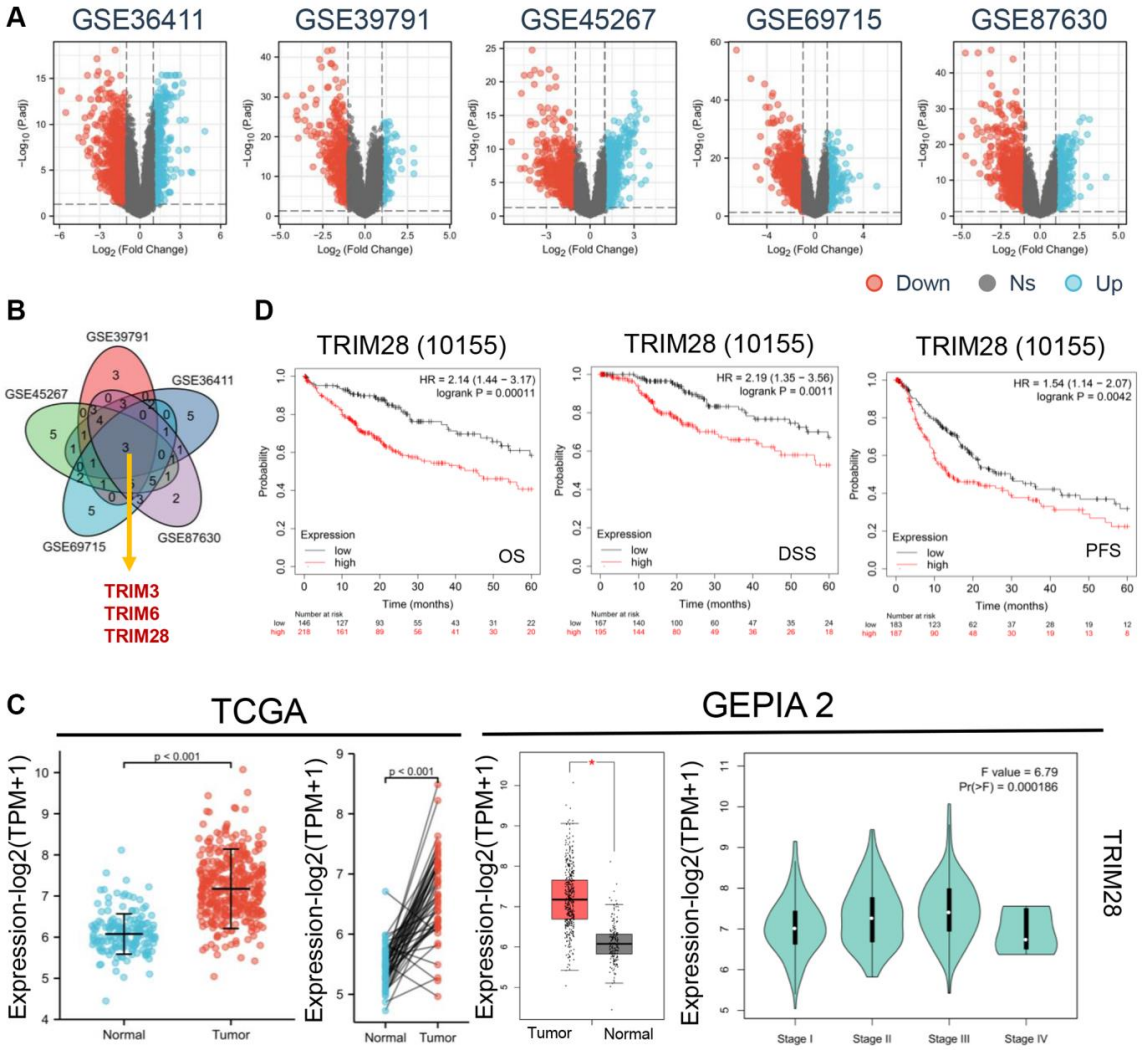
**The expression levels of TRIMs protein with clinical features in HCC.** (**A**) Differential gene expression in HCC and normal tissues from GSE36411, GSE39791, GSE45267, GSE69715 and GSE87630 (blue: overexpression, red: down expression). (**B**) Venny diagrams was used to take the intersection three genes (TRIM3, TRIM6 and TRIM28) from above five datasets. (**C**) We analyzed the expression level of TRIM28 total protein between normal tissue and HCC tissue from TCGA, *p* < 0.001 (left). Based on the GEPIA 2 data, the TRIM28 expression levels was analyzed by main pathological stages (stage I, stage II, stage III, and stage IV) (right). Log2 (TPM + 1) was applied for log-scale. ^*^p < 0.05. (**D**) Relationship between TRIM28 expression and OS, DSS and PFS by Kaplan-Meier Plotter in HCC from TCGA databases.

To compare the expression levels of TRIMs in HCC, five subsets of microarray data were extracted from the GEO database, including GSE39791 (Platform: GPL10558), GSE36411 (Platform: GPL10558), GSE45267 (Platform: GPL570), GSE69715 (Platform: GPL570) and GSE87630 (Platform: GPL6947) (Figure 1A). Principal Component analysis was then performed, which showed that the quality and feasibility of the data met the criterion for further analyses (Supplementary Figure 1A). By taking the intersection of these five databases, three potential TRIMs, TRIM3, TRIM6 and TRIM28 (*P* < 0.01) (Figure 1B) were found to be significantly higher in HCC tissues. Data from Oncomine database showed that TRIM6 and TRIM28 expression was significantly higher in HCC compared with that in non-cancerous liver tissues (Supplementary Figure 1B).

Subsequently, we assessed the TRIM3, TRIM6 and TRIM28 expression profile in normal and HCC tissues collected from TCGA and GEPIA2 dataset cohorts, where the results indicated that TRIM6 and TRIM28 expression was significantly increased in HCC tissues compared with that in normal tissues according to the TCGA database (Figure 1C, Supplementary Figure 1C). However, only the expression of TRIM28 was significantly higher in GEPIA2 (*P* < 0.05). In addition, significant differences were also found among the TRIM28 levels and tumor staging groups (*P* = 0.000186) according to the GEPIA2 datasets (Figure 1C). Therefore, we then focused on TRIM28 for further analyses.

As shown in Figure 1D, patients with HCC expressing higher levels of TRIM28 were associated with reduced OS, DSS and PFS compared with those with lower TRIM28 expression levels. Subsequently, data containing the required clinical characteristics of 374 patients with HCC were acquired from TCGA. TRIM28 overexpression was found to associate significantly with age, BMI, pathological staging, histological grade, α-foetalprotein (ng/ml) and higher TNM staging. The detailed clinicopathological features are shown in Table 1. These results suggest that TRIM28 can contribute to HCC progression.

**Table 1:** Correlation between TRIM28 expression and clinicopathological characteristics of HCCs.

**Characteristic**	**Levels**	**Overall**	**TRIM28**		***p***	**Statistic**	**Method**
			**Low expression**	**High expression**			
*n*		374	187	187			
T stage, *n* (%)	T1	183 (49.3%)	112 (30.2%)	71 (19.1%)	<0.001	18.9	Chisq.test
	T2	95 (25.6%)	37 (10%)	58 (15.6%)			
	T3	80 (21.6%)	30 (8.1%)	50 (13.5%)			
	T4	13 (3.5%)	6 (1.6%)	7 (1.9%)			
N stage, *n* (%)	N0	254 (98.4%)	118 (45.7%)	136 (52.7%)	0.627		Fisher.test
	N1	4 (1.6%)	1 (0.4%)	3 (1.2%)			
M stage, *n* (%)	M0	268 (98.5%)	124 (45.6%)	144 (52.9%)	1.000		Fisher.test
	M1	4 (1.5%)	2 (0.7%)	2 (0.7%)			
Pathologic stage, *n* (%)	Stage I	173 (49.4%)	104 (29.7%)	69 (19.7%)	<0.001		Fisher.test
	Stage II	87 (24.9%)	36 (10.3%)	51 (14.6%)			
	Stage III	85 (24.3%)	31 (8.9%)	54 (15.4%)			
	Stage IV	5 (1.4%)	3 (0.9%)	2 (0.6%)			
Tumor status, *n* (%)	Tumor free	202 (56.9%)	108 (30.4%)	94 (26.5%)	0.146	2.11	Chisq.test
	With tumor	153 (43.1%)	69 (19.4%)	84 (23.7%)			
Gender, *n* (%)	Female	121 (32.4%)	56 (15%)	65 (17.4%)	0.377	0.78	Chisq.test
	Male	253 (67.6%)	131 (35%)	122 (32.6%)			
Age, *n* (%)	≤60	177 (47.5%)	75 (20.1%)	102 (27.3%)	0.006^**^	7.54	Chisq.test
	>60	196 (52.5%)	112 (30%)	84 (22.5%)			
BMI, *n* (%)	≤25	177 (52.5%)	78 (23.1%)	99 (29.4%)	0.034^*^	4.51	Chisq.test
	>25	160 (47.5%)	90 (26.7%)	70 (20.8%)			
Residual tumor, *n* (%)	R0	327 (94.8%)	164 (47.5%)	163 (47.2%)	1.000		Fisher.test
	R1	17 (4.9%)	9 (2.6%)	8 (2.3%)			
	R2	1 (0.3%)	1 (0.3%)	0 (0%)			
Histologic grade, *n* (%)	G1	55 (14.9%)	34 (9.2%)	21 (5.7%)	<0.001	18.11	Chisq.test
	G2	178 (48.2%)	103 (27.9%)	75 (20.3%)			
	G3	124 (33.6%)	45 (12.2%)	79 (21.4%)			
	G4	12 (3.3%)	4 (1.1%)	8 (2.2%)			
Adjacent hepatic tissue inflammation, *n* (%)	None	118 (49.8%)	74 (31.2%)	44 (18.6%)	0.126	4.15	Chisq.test
	Mild	101 (42.6%)	50 (21.1%)	51 (21.5%)			
	Severe	18 (7.6%)	9 (3.8%)	9 (3.8%)			
AFP (ng/ml), *n* (%)	≤400	215 (76.8%)	135 (48.2%)	80 (28.6%)	<0.001	37.57	Chisq.test
	>400	65 (23.2%)	12 (4.3%)	53 (18.9%)			
Albumin (g/dl), *n* (%)	<3.5	69 (23%)	39 (13%)	30 (10%)	0.830	0.05	Chisq.test
	≥3.5	231 (77%)	125 (41.7%)	106 (35.3%)			
Prothrombin time, *n* (%)	≤4	208 (70%)	111 (37.4%)	97 (32.7%)	0.619	0.25	Chisq.test
	>4	89 (30%)	51 (17.2%)	38 (12.8%)			
Child-Pugh grade, *n* (%)	A	219 (90.9%)	121 (50.2%)	98 (40.7%)	0.726		Fisher.test
	B	21 (8.7%)	10 (4.1%)	11 (4.6%)			
	C	1 (0.4%)	1 (0.4%)	0 (0%)			
Vascular invasion, *n* (%)	No	208 (65.4%)	116 (36.5%)	92 (28.9%)	0.241	1.37	Chisq.test
	Yes	110 (34.6%)	53 (16.7%)	57 (17.9%)			
Fibrosis ishak score, *n* (%)	0	75 (34.9%)	48 (22.3%)	27 (12.6%)	0.076	6.88	Chisq.test
	1/2	31 (14.4%)	16 (7.4%)	15 (7%)			
	3/4	28 (13%)	10 (4.7%)	18 (8.4%)			
	5/6	81 (37.7%)	46 (21.4%)	35 (16.3%)			
OS event, *n* (%)	Alive	244 (65.2%)	132 (35.3%)	112 (29.9%)	0.039^*^	4.26	Chisq.test
	Dead	130 (34.8%)	55 (14.7%)	75 (20.1%)			
DSS event, *n* (%)	Alive	287 (78.4%)	147 (40.2%)	140 (38.3%)	0.336	0.92	Chisq.test
	Dead	79 (21.6%)	35 (9.6%)	44 (12%)			
PFI event, *n* (%)	Alive	191 (51.1%)	102 (27.3%)	89 (23.8%)	0.214	1.54	Chisq.test
	Dead	183 (48.9%)	85 (22.7%)	98 (26.2%)			

^*^p < 0.05, ^**^p < 0.01. Abbreviations: Fisher.test: fisher exact test; Chisq.test: Chi-square test.

### Relationship between methylation status and TRIM28 expression in HCC

DNA methylation is as an important mechanism of epigenetic modification that serves an important role in regulating gene expression. Since TRIM28 expression was found to be increased in HCC, we then assessed whether DNA methylation contributed to the regulation of TRIM28 expression. To assess the relationship between methylation status and TRIM28 expression in HCC, we first visualized 12 methylation sites (cg05663122, cg05025162, cg26981251, cg24109975, cg12528394, cg01339029, cg11909976, cg19476058, cg17840453, cg18397137, cg06180363 and cg05678175) in the DNA sequences of TRIM28, which were identified using MethSurv (Supplementary Table 1, Supplementary Figure 2A). We found that there was a negative correlation between TRIM28 expression and the methylation status of three methylation sites, cg11909976, cg19476058 and cg05678175 (Supplementary Table 1). Among these sites, only the methylation level of cg05678175 was significantly decreased in HCC (Supplementary Figure 2A). As shown in Supplementary Figure 2B, we found that higher TRIM28 expression was associated with its hypomethylation. Subsequent survival analyses also revealed that the hypomethylation of cg05678175 was positively associated with the OS of patients with HCC. These results suggest that cg05678175 is a potential functional methylation site on the *TRIM28* gene.

### Relationship between the immune state and TRIM28 expression in HCC

Alterations of immune states have been suggested to be a typical feature in patients with HCC. Since TRIM28 was shown to be associated with the prognosis of HCC, we next investigated whether this phenomenon was at least partially attributed to changes to immune states. Using TCGA, we obtained the expression profiles of a series of genes associated with immune checkpoints that were found to be associated with TRIM28. As shown in Supplementary Table 2, programmed cell death protein-1 (PD-1), cytotoxic T lymphocyte antigen 4 (CTLA-4), T cell immunoglobulin and mucin domain-containing-3 (TIM3), T cell immunoreceptor with Ig and ITIM domains (TIGIT), CD8B, CD27, CD96, CD40 ligand (CD40LG) and TNF receptor superfamily member 4 (TNFRSF4) expression levels were upregulated, whilst adenosine A2a receptor (ADORA2A), CD33, killer cell lectin like receptor C1 (KLRC1), lymphocyte Activating 3 (LAG3), programmed death-ligand (PD-L1), Siglec 7 and Siglec 9 expression was downregulated in HCC. The values of these genes in predicting the various different survival outcome parameters are also shown in Supplementary Table 2. The general landscape showing the relationship between TRIM28 expression and immune checkpoint gene alterations in HCC was concisely visualized, including fusion, amplification, deep deletion, truncating and missense mutations (Supplementary Figure 3). The detailed relationship between TRIM28 expression and each representative immune checkpoint is individually presented in Supplementary Table 3. Of note, alterations in TRIM28 expression showed statistically significant co-occurrence instead of mutual exclusivity with a substantial quantity of immune checkpoints, such as Siglec 7, CD33 and Siglec 9.

**Figure 2:**
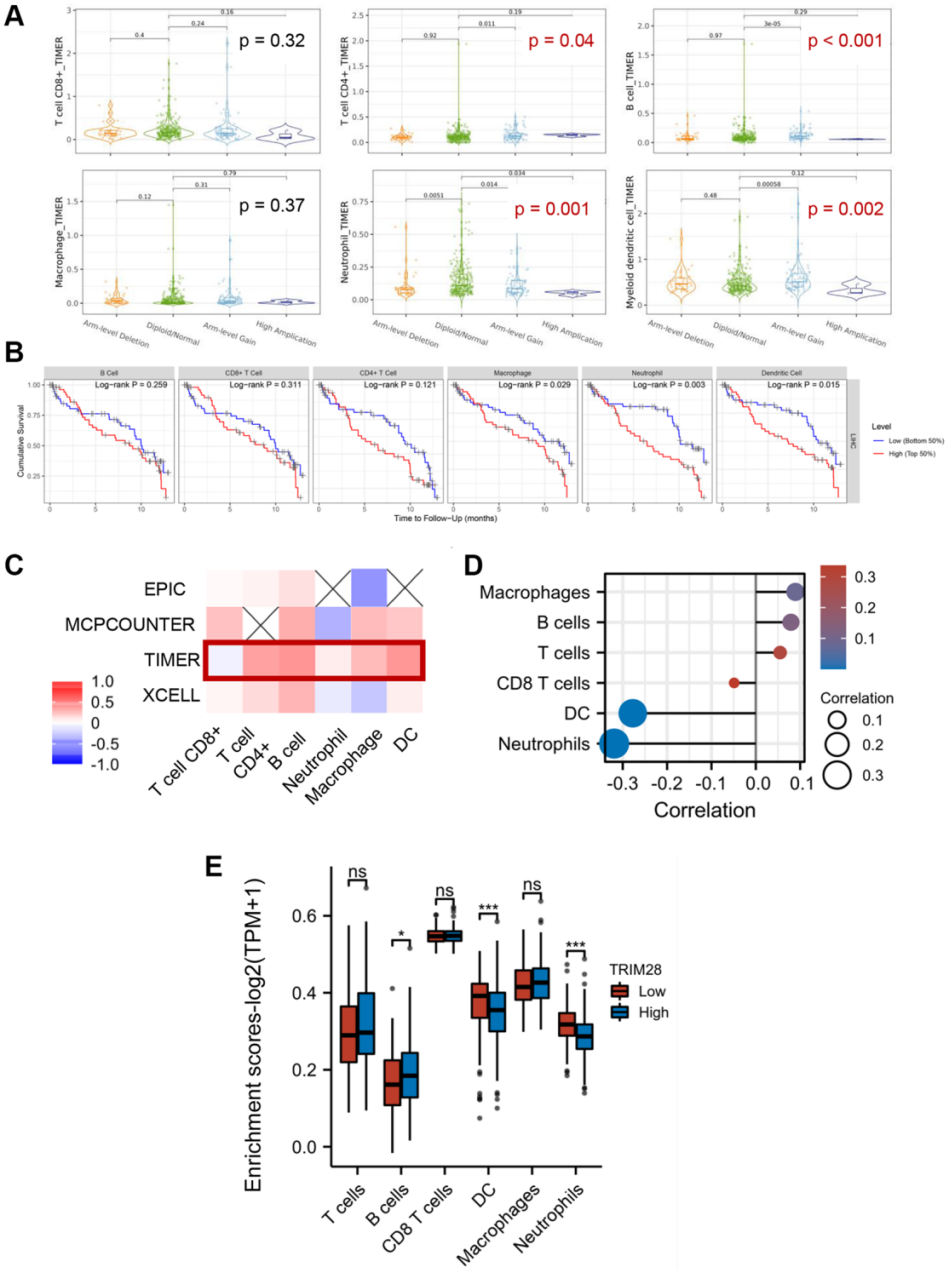
**Correlation between immune cell infiltration and TRIM28 in HCC.** (**A**) The quantity of immune cells (T cell CD8, T cell CD4, B cell, Macrophage, Neutrophil and Myeloid dendritic cell) in HCC with altered TRIM28 copy numbers from TIMER 2.0 database. (**B**) Kaplan-Meier plots were used to analyze the TRIM28-binding immune infiltration and overall survival rate of HCC. Among these, T cell CD4 (0.036), Macrophage (0.007) and Neutrophil (0.01) markedly positively correlated with infiltrating levels. (**C**, **D**). Immune cells levels were showed in a heatmap and lollipop diagrams from TCGA. (**E**) The immune genes associated TRIM28 expression level analysis from TCGA. Log2 (TPM + 1) was applied for log-scale. ^*^p < 0.05; ^***^p < 0.001. Abbreviation: ns: no significant.

Next, we conducted a series of gene expression analyses on the TIMER 2.0 database to evaluate the potential relationship between TRIM28 expression and immune infiltration in HCC. The analyses showed that alterations in TRIM28 copy numbers were associated with the degree of infiltration by several immune cell types, including CD4+ T cells, B cells, neutrophil and myeloid dendritic cells in HCC (Figure 2A). In addition, we further analysed the impact of immune infiltration on the clinical prognosis of patients with HCC, which showed that increased filtration by CD4+ T cells, macrophages and neutrophils were associated with a poorer prognosis in patients with HCC. Their survival period was shown to be <24 months (Figure 2B).

We next evaluated the correlation between TRIM28 expression and macrophage, T cells, B cells, neutrophil or dendritic cell infiltration using TIMER and TCGA. The results showed that the expression of TRIM28 was positively correlated with macrophage, T cells and B cell infiltration according to both TCGA and TIMER databases (Figure 2C, 2D). To further explore the relationship between TRIM28 and immune-infiltrating cells, we also validated the association of TRIM28 with a set of immune biomarkers representing seven different immune cell types. CD8A, CD8B, CD19, CD79A, interferon regulatory factor 5, integrin subunit α M, human leukocyte antigen (HLA)-DPB1, HLA-DQB1, HLA-DRA, HLA-DPA1, CD1C, neuropilin 1 and integrin subunit αX were found to be significantly correlated with TRIM28 expression in HCC (Table 2). We also performed enrichment analysis in the TCGA database using seGSEA, which found that increased B cell infiltration was observed in HCC with higher levels of TRIM28 expression (Figure 2E). Taken together, tumor-infiltrating immune cells, especially B cells, may serve influential roles in the clinical outcomes of patients with HCC with different levels of TRIM28 expression.

**Table 2:** Correlation between TRIM28 and Biomarker of HCC.

**Immune cell**	**Biomarker**	**R-value**	***P*-value**
CD8+ T cell	CD8A	0.16	0.0022
	CD8B	0.21	<0.001
CD4+ T cell	CD4	0.092	0.079
B cell	CD19	0.26	<0.001
	CD79A	0.21	<0.001
M1 macrophage	NOS2	−0.12	0.02
	IRF5	0.31	<0.001
	PTGS2	0.096	0.065
	CD80	0.018	0.734
M2 macrophage	CD163	0.053	0.31
	VSIG4	0.062	0.24
	MS4A4A	0.054	0.3
Neutrophil	CEACAM8	0.046	0.38
	ITGAM	0.2	<0.001
	CCR7	0.065	0.21
Dendritic cell	HLA-DPB1	0.19	<0.001
	HLA-DQB1	0.16	0.002
	HLA-DRA	0.14	0.008
	HLA-DPA1	0.13	0.013
	CD1C	0.2	<0.001
	NRP1	0.3	<0.001
	ITGAX	0.29	<0.001

### TRIM28/H2AX/CDK4 axis is involved in the poor prognosis of patients with HCC

**Figure 3:**
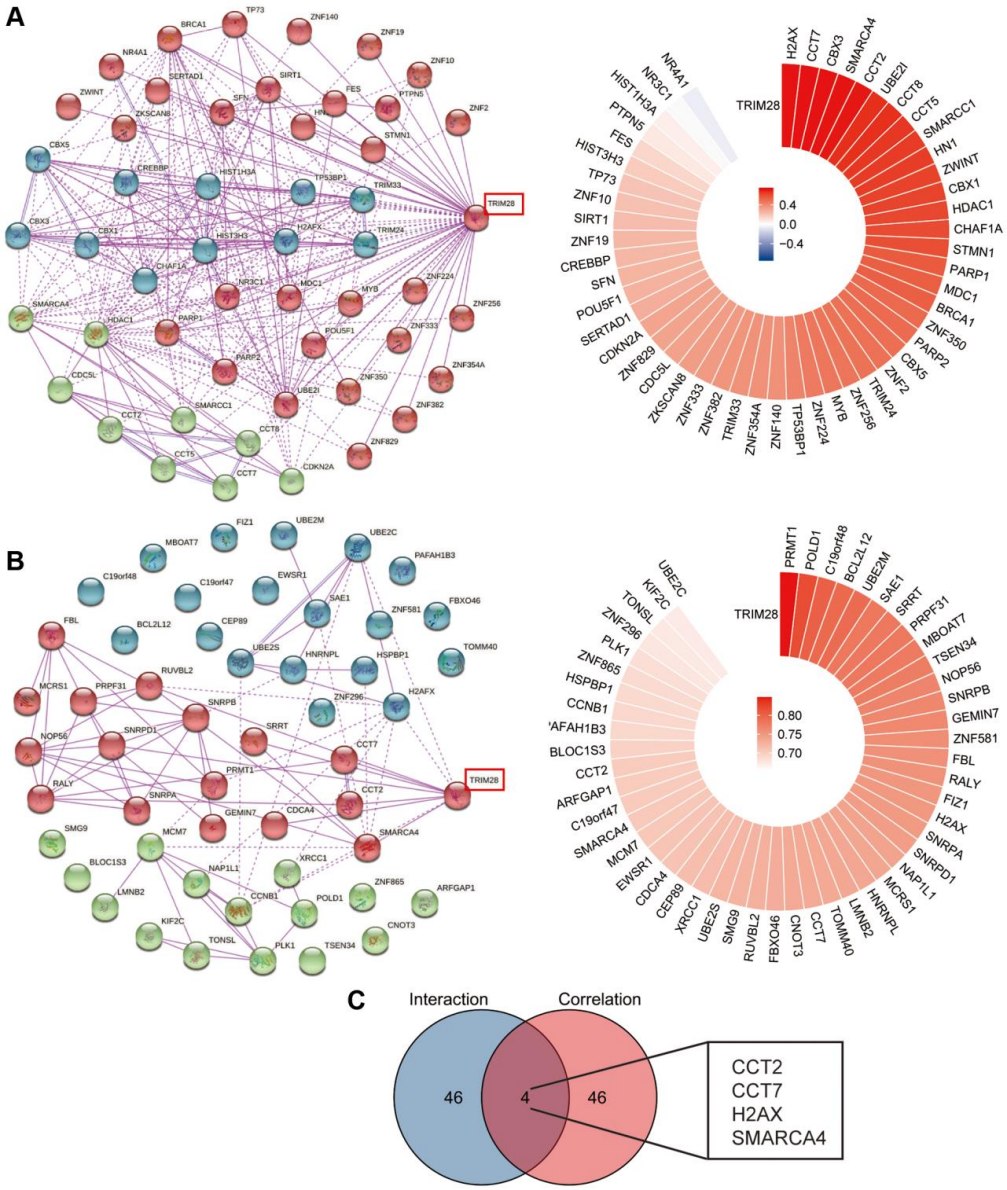
**TRIM28-related interaction and correlation gene enrichment analysis in HCC.** (**A**, **B**) We obtained the TRIM28-binding top 50 proteins using the STRING tool (three different colors using Kmeans clustering), (**A**) the TRIM28-binding interaction genes from cBioPortal data, (**B**) the TRIM28-binding correlation genes from Gephia 2 data. The corresponding circos plot data in the detailed genes are displayed. (**C**) Venn diagrams was used to conduct the interaction analysis of the TRIM28-binding and correlated four genes (CCT2, CCT7, H2AX and SMARCA4).

To predict the potential function of TRIM28, we performed interaction and correlation analyses between TRIM28 expression and other candidate genes in HCC using the cBioPortal and GEPIA2 databases. We conducted a joint analysis in the high-TRIM28 and low-TRIM28 expression groups. The top 50 genes showing positive association with TRIM28 expression were presented in a heatmap and a circos plot as three clusters using Kmeans clustering (Figure 3A, 3B and Supplementary Figure 4A). We subsequently found four candidate genes, chaperonin containing TCP1 subunit (CCT)2, CCT7, H2AX and SWI/SNF-related, matrix-associated, actin dependent regulator of chromatin (SMARC), subfamily A, member 4, from the intersection of the top 50 genes (Figure 3C).

**Figure 4:**
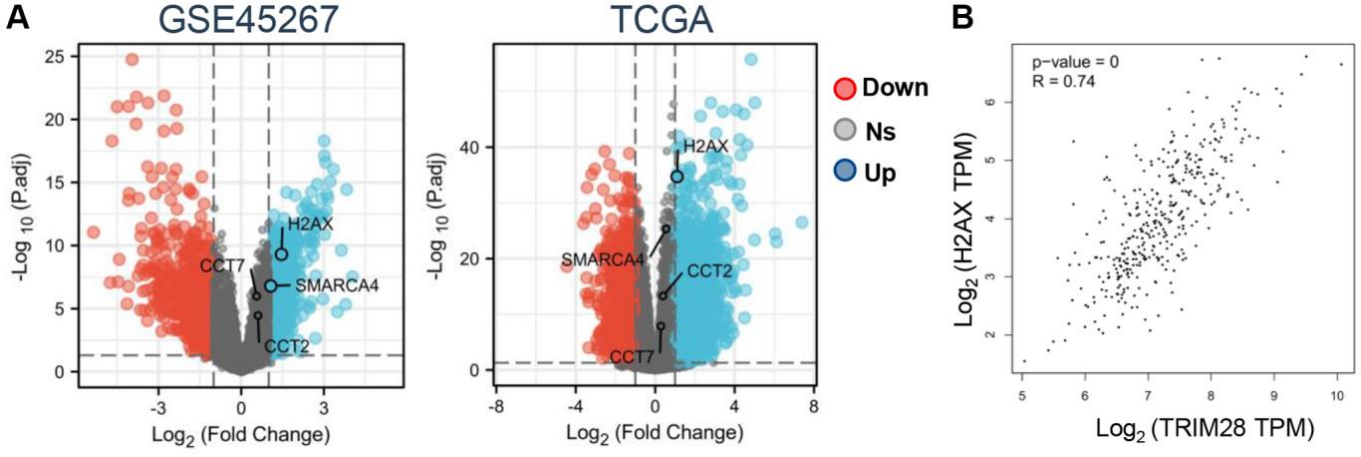
**Correlation between TRIM28/H2AX/CDK4 and clinicopathological characteristics of HCC.** (**A**) An overview of CCT2, CCT7, H2AX and SMARCA4 expression levels in HCC from GSE45267 and TCGA (blue: overexpression, red: down expression, Abbreviation: ns: no significant. (**B**) Correlation analysis between TRIM28 and H2AX expression in HCC from TCGA data.

As a result, we analyzed the expression profile of CCT2, CCT7, H2AX and SMARCA4 from the GSE39791, GSE36411, GSE45267, GSE69715 and GSE87630 datasets. H2AX and SMARCA4 expression was significantly higher according to the GSE45267 dataset (Figure 4A), but there were no differences in the GSE39791, GSE36411, GSE69715 and GSE87630 datasets (Supplementary Figure 4A). In order to find the target gene of TRIM28 more accurately, we also analyzed the expression data of CCT2, CCT7, H2AX and SMARCA4 in a dataset obtained from TCGA, which found that only H2AX expression was upregulated in HCC (Figure 4A). Subsequently, we also confirmed that there was a strongly positive correlation between TRIM28 and H2AX expression in HCC according to the TCGA dataset (Figure 4B). To evaluate the value of TRIM28 or H2AX expression for predicting the prognosis of patients with cancer, the association between TRIM28 or H2AX expression and OS, PFS and DSS were analyzed in the same Kaplan Meier-Plotter cohort. Higher TRIM28 or H2AX expression was significantly associated with decreased OS, RFS, PFS and DSS in patients with HCC (Figure 1D, Supplementary Figure 4B). These results suggest that the TRIM28/H2AX axis is involved in HCC tumor progression to result in the poor prognosis of patients with HCC.

To further unravel the downstream signaling pathways of TRIM28 and H2AX, we used cBioPortal and found that the p53, TGF-β, Wnt, mitogen-activated protein kinase and oxytocin receptor-mediated signaling pathways were associated with TRIM28 and H2AX expression (Supplementary Figure 5A). By comparing the top five genes that were associated with TRIM28 and H2AX expression, we found that four genes, CDK4, SMARCD1, SMARCB1 and Bcl-2-associated X, apoptosis regulator (BAX), were potentially correlated with TRIM28 and H2AX expression (Supplementary Figure 5B). We first found that high levels of CDK4, SMARCD1, SMARCB1 and BAX expression were associated with a reduced OS in patients with HCC (Supplementary Figure 6A). Using Spearman’s correlation analysis, we noted that there was a strong correlation between TRIM28 or H2AX expression and the expression these four candidate genes in HCC according to TCGA (Supplementary Figure 6B). We then analyzed the association between the expression of these genes and patient prognosis using univariate or multivariate Cox analysis in patients with HCC (Tables 3.1–3.3 and Supplementary Tables 4–15). Univariate Cox analysis demonstrated that higher expression levels of CDK4, SMARCD1, SMARCB1 and BAX were significantly associated with reduced OS. However, only the expression of three genes (CDK4, SMARCD1 and SMARCB1) were significantly associated with DSS and only CDK4 and SMARCD1 were significantly associated with PFS (Supplementary Tables 1–12). Multivariate Cox analysis confirmed that CDK4 overexpression was an independent risk factor for reduced survival in patients with HCC (Tables 3.1–3.3). Therefore, we speculated that the TRIM28/H2AX/CDK4 axis may serve a crucial role in HCC.

**Table 3.1.** Univariate and multivariate Cox proportional hazards analysis of TRIM28 expression and OS for patients with HCC.

**Characteristics**	**Total (*N*)**	**Univariate analysis**		**Multivariate analysis**	
		**HR (95% CI)**	***P*-value**	**HR (95% CI)**	***P*-value**
T stage (T2 & T3 & T4 vs. T1)	370	2.126 (1.481–3.052)	**<0.001**	0.489 (0.062–3.894)	0.500
Pathologic stage (Stage II & Stage III & Stage IV vs. Stage I)	349	2.090 (1.429–3.055)	**<0.001**	3.710 (0.455–30.223)	0.221
Age (>60 vs. ≤60)	373	1.205 (0.850–1.708)	0.295	1.579 (0.970–2.571)	0.066
BMI (>25 vs. ≤25)	336	0.798 (0.550–1.158)	0.235	1.143 (0.703–1.859)	0.590
Histologic grade (G3 & G4 vs. G1 & G2)	368	1.091 (0.761–1.564)	0.636	1.513 (0.897–2.550)	0.120
AFP(ng/ml) (>400 vs. ≤400)	279	1.075 (0.658–1.759)	0.772	0.730 (0.392–1.359)	0.321
TRIM28 (High vs. Low)	373	1.591 (1.122–2.257)	**0.009**	1.198 (0.632–2.269)	0.580
H2AX (High vs. Low)	373	1.519 (1.073–2.151)	**0.019**	0.801 (0.430–1.492)	0.484
CDK4 (High vs. Low)	373	1.921 (1.350–2.733)	**<0.001**	1.908 (1.060–3.437)	**0.031**

**Table 3.2.** Univariate and multivariate Cox proportional hazards analysis of TRIM28 expression and PFS for patients with HCC.

**Characteristics**	**Total (*N*)**	**Univariate analysis**		**Multivariate analysis**	
		**HR (95% CI)**	***P*-value**	**HR (95% CI)**	***P*-value**
T stage (T2 & T3 & T4 vs. T1)	362	2.829 (1.747–4.582)	**<0.001**	0.254 (0.029–2.196)	0.213
Pathologic stage (Stage II & Stage III & Stage IV vs. Stage I)	341	2.909 (1.718–4.925)	**<0.001**	10.404 (1.138–95.097)	0.038
Age (>60 vs. ≤60)	365	0.846 (0.543–1.317)	0.458	0.950 (0.512–1.764)	0.872
BMI (>25 vs. ≤25)	329	0.826 (0.512–1.330)	0.431	1.528 (0.807–2.895)	0.193
Histologic grade (G3 & G4 vs. G1 & G2)	360	1.086 (0.683–1.728)	0.726	1.411 (0.712–2.796)	0.324
AFP(ng/ml) (>400 vs. ≤400)	275	0.867 (0.450–1.668)	0.668	0.486 (0.201–1.176)	0.109
TRIM28 (High vs. Low)	365	1.525 (0.977–2.380)	0.063	1.136 (0.503–2.565)	0.759
H2AX (High vs. Low)	365	1.396 (0.897–2.175)	0.140	0.485 (0.213–1.105)	0.085
CDK4 (High vs. Low)	365	2.346 (1.479–3.721)	**<0.001**	3.893 (1.793–8.451)	**<0.001**

**Table 3.3.** Univariate and multivariate Cox proportional hazards analysis of TRIM28 expression and DSS for patients with HCC.

**Characteristics**	**Total (*N*)**	**Univariate analysis**		**Multivariate analysis**	
		**HR (95% CI)**	***P*-value**	**HR (95% CI)**	***P*-value**
T stage (T2 & T3 & T4 vs. T1)	370	2.360 (1.745–3.191)	**<0.001**	0.457 (0.059–3.510)	0.452
Pathologic stage (Stage II & Stage III & Stage IV vs. Stage I)	349	2.284 (1.670–3.122)	**<0.001**	4.388 (0.568–33.927)	0.156
Age (>60 vs. ≤60)	373	0.960 (0.718–1.284)	0.783	1.040 (0.723–1.494)	0.834
BMI (>25 vs. ≤25)	336	0.936 (0.689–1.272)	0.673	1.103 (0.758–1.605)	0.608
Histologic grade (G3 & G4 vs. G1 & G2)	368	1.152 (0.853–1.557)	0.355	1.056 (0.715–1.559)	0.785
AFP (ng/ml) (>400 vs. ≤400)	279	1.045 (0.698–1.563)	0.832	0.833 (0.519–1.338)	0.449
TRIM28 (High vs. Low)	373	1.393 (1.041–1.864)	**0.026**	0.880 (0.551–1.405)	0.592
H2AX (High vs. Low)	373	1.543 (1.153–2.066)	**0.004**	0.876 (0.552–1.391)	0.576
CDK4 (High vs. Low)	373	1.807 (1.348–2.424)	**<0.001**	2.067 (1.341–3.185)	**<0.001**

### Construction and validation of the TRIM28/H2AX/CDK4 diagnostic model in HCC

**Figure 5:**
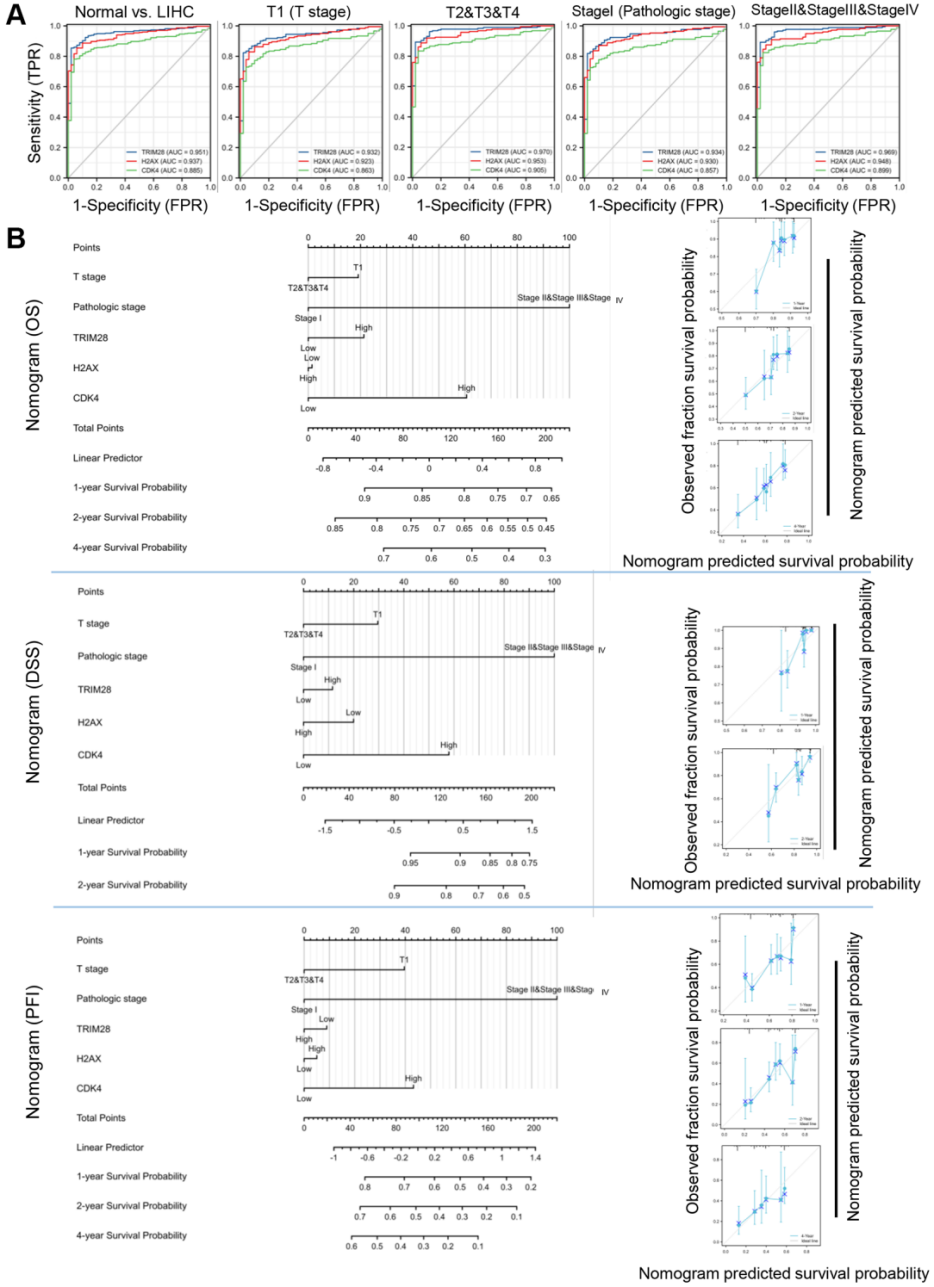
**Diagnostic and prognostic values of TRIM28, H2AX and CDK4 expression in HCC.** (**A**) The AUC for ROC curves for TRIM28, H2AX and CDK4 in normal liver tissues and HCC tissues, subgroup analysis for T1 stage, T2/T3/T4 stage, Pathologic stage I and Pathologic stage II/III/IV were computed. (**B**) Nomogram for predicting probability of patients with 1-, 3- and 5-year OS, DSS, PFS in entire TCGA cohort in HCC. Calibration curves of nomogram on consistency between predicted and observed 1-, 3-, and 5-year survival in entire TCGA cohort. Dashed line at 45° implicated a perfect prediction, and the actual performances of our nomogram were shown in blue lines.

We conducted Receiver Operating Characteristic (ROC) curve analysis of TRIM28, H2AX and CDK4 to evaluate their diagnostic value in patients with HCC. The Area under the receiver operating characteristic curve (AUC) was calculated to be 0.945, 0.945 and 0.940 for TRIM28, H2AX and CDK4, respectively (Figure 5A). Subgroup analysis also demonstrated the diagnostic value of TRIM28/H2AX/CDK4 in various important clinical characteristics of HCC, with AUC values of 0.925, 0.932 and 0.929 for T1, respectively; 0.965, 0.959 and 0.952 for T2/3/4, respectively; 0.926, 0.938 and 0.926 for stage I, respectively; and 0.963, 0.954 and 0.949 for stage II/III/IV, respectively (Figure 5A).

Based on clinicopathological factors, we constructed a prognostic nomogram with the aim of providing a quantitative analytical tool that can be used to predict the 1-, 2-, or 4-year OS, DSS and PFS rate of individual patients by combining the expression levels of TRIM28/H2AX/CDK4 in the entire TCGA cohort. The calibration curves of the nomogram showed consistencies between the predicted and observed OS/DSS/PFS probabilities (Figure 5B).

**Figure 6:**
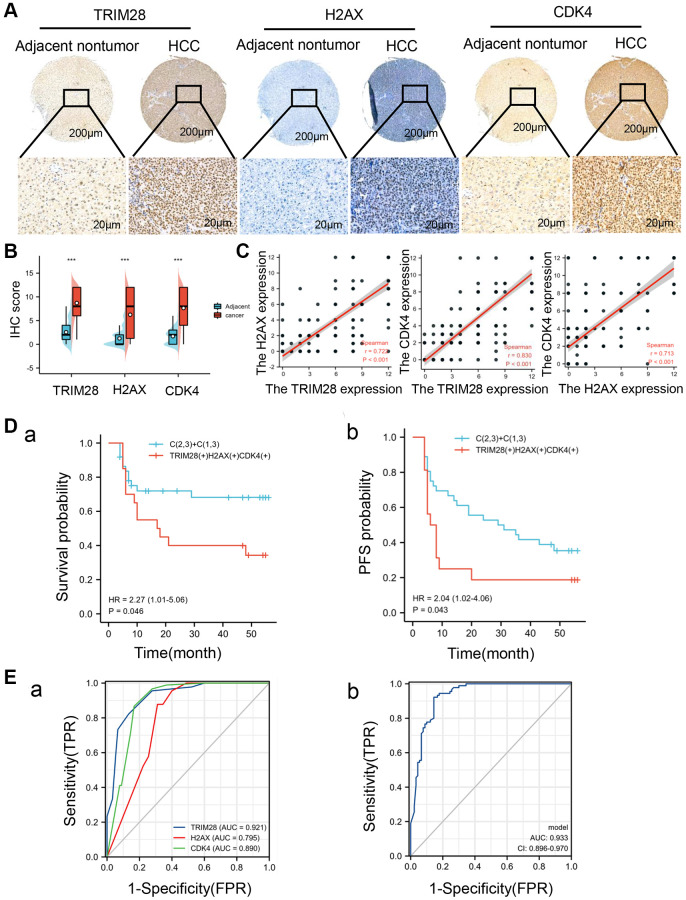
**High TRIM28, H2AX, and CDK4 expression is associated with poor prognosis in patients with HCC.** (**A**) An Immunohistochemical analysis of TRIM28, H2AX and CDK4 expression in HCC tissues and adjacent non-tumorous tissues (*n* = 90). (**B**) The expression of TRIM28, H2AX and CDK4 in HCC and corresponding adjacent nontumor tissues. ^***^p < 0.001. (**C**) The association between the expression of TRIM28, H2AX and CDK4 in HCC patients. (**D** (**a**, **b**)) A Kaplan-Meier analysis of TRIM28, H2AX and CDK4 expression those three genes co-expression for overall survival and PFS in an independent cohort 90 HCC patients. (**E** (**a**, **b**)). The AUC for ROC curve analyses for TRIM28 (AUC = 0.921), H2AX (AUC = 0.795), CDK4 (AUC = 0.890) expression and three gene co-expression (AUC = 0.933) in our independent cohort of HCC.

To comprehensively evaluate the clinical significance of TRIM28, H2AX and CDK4 expression in patients with HCC, we analyzed the expression levels of TRIM28, H2AX and CDK4 in a set of HCC tissue microarrays using IHC staining. In 90 pairs of primary HCC tissue samples and corresponding adjacent non-cancerous tissue samples, the staining intensities of TRIM28, H2AX and CDK4 were found to be significantly higher in the HCC tissues (Figure 6A, 6B). Additionally, we found that TRIM28 is positively correlated with H2AX and CDK4 expression (Figure 6C), which is similar to the results obtained using data from TCGA. In these 90 HCC cases containing the clinicopathological information, the staining intensities of TRIM28, H2AX and CDK4 in tissues from late-stage HCC (stage III) were stronger compared with those from early-stage HCC (stage I and II). In addition, their expression in tissues at stages II and III according to American Joint Committee on Cancer were higher compared with those at stage I. Their expression levels were also found to associate with T, N and M staging (Supplementary Tables 16–18). Univariate analysis showed that TRIM28, H2AX and CDK4 expression were significant independent factors for OS and PFS (Supplementary Tables 19 and 20). Subsequently, multivariate analysis revealed that the expression of CDK4 was an independent predictor for OS and PFS in HCC (Supplementary Tables 16 and 17). Kaplan-Meier survival analysis suggested that higher expression levels of TRIM28, H2AX or CDK4 expression were associated with poorer prognoses in patients with HCC (Supplementary Figure 7A, 7B). The high expression levels of all three proteins combined was associated with reduced OS compared with higher expression levels of either of the three proteins alone, whereas higher PFS rates were observed in patients that did not express high levels of these three proteins (Figure 6D (a, b)). As shown in Table 4, high expression levels of TRIM28, H2AX or CDK4 were significantly associated with tumor recurrence in an independent cohort of HCC. To detect the diagnostic value of TRIM28, H2AX and CDK4 in patients with HCC, ROC analysis was performed using the “roc” function in the pROC R package (Version 1.17.0.1) and “ggplot2” R package (Version 3.3.3). In the 90 HCC cases, the AUC was calculated to be 0.921, 0.795 and 0.890, respectively (Figure 6E (a)), which was consistent with the results from TCGA. The AUC of the integrated analysis of these three targets was 0.933, which was higher compared with either of the three proteins alone (Figure 6E (b)). These results implicate the potential roles of TRIM28, H2AX and CDK4 in evaluating the prognosis of patients with HCC.

**Table 4:** Correlation between genes expression and recurrence in HCC.

**Gene expression**	**Microarray findings (*n* = 90)**		***P*-value**	**Methods**
	**Reoccurrence**	**Nonrecurrences**		
TRIM28			0.047	Chisq.test
High	22	29		
Low	9	30		
H2AX			0.038	Chisq.test
High	32	20		
Low	15	23		
CDK4			0.031	Chisq.test
High	23	28		
Low	9	30		

Abbreviation: Chisq.test: Chi-square test.

## DISCUSSION

In the present study, we performed a systemic analysis of TRIMs, an important subgroup of E3 ubiquitin ligases, in HCC, which found that TRIM28 may function as a key regulator through its downstream targets H2AX and CDK4. In addition, we evaluated the diagnostic values of TRIM28, H2AX and CDK4 and established a nomogram to predict the clinical outcomes of patients with HCC. These results suggest that the TRIM28/H2AX/CDK4 axis can serve as a potential biomarker and therapeutic target of HCC.

TRIM28, also known as KRAB-associated protein 1, is a member of the TRIM transcription factor family. In addition, it is a specific SUMO E3 ligase that can regulate autophagy, immunity and carcinogenesis [5, 16]. A previous study by Wang et al. [14] reported that TRIM28 overexpression can promote HCC cell proliferation, which is significantly associated with tumor staging in patients with HCC [14]. In addition, another previous report showed that the TRIM28-melanoma antigen complex can promote the Warburg effect and HCC progression by targeting fructose-bisphosphatase 1 for ubiquitination and degradation [17]. TRIM28 can also interact with the ubiquitin-conjugating enzyme E2S to accelerate cell cycle progression by regulating p27 ubiquitination [18]. Similar functions of TRIM28 were found in animal models of HCC, where TRIM28 was found to accelerate histone deacetylase 6 ubiquitination and degradation [19]. By contrast, transcription cofactors TRIM24, TRIM28 and TRIM33 can form regulatory complexes to suppress the progression of HCC [20]. Therefore, the function and potential mechanism of TRIM28 in HCC progression remain largely unclear. In this study, we identified that nuclear TRIM28 expression was significantly higher in HCC tissues, which was in turn was closely associated with the clinical outcomes of patients with HCC. Using bioinformatics enrichment analysis and validation, H2AX and CDK4 were found to be the functional targets of TRIM28, revealing the possible mechanism of TRIM28 in HCC. In addition, the novel, possibly functional cg05678175 methylation site in the *TRIM28* gene was identified for the first time. Our results revealed that the methylation level of this site of the *TRIM28* gene was significantly decreased in HCC, which was associated with a poorer prognosis in patients with HCC. These data suggest that cg05678175 may be a potentially functional methylation site of TRIM28.

Immunotherapy targets the immune system to damage and destroy tumors in the tumor microenvironment (TME), which has been an effective approach for tumor management [21]. Immune checkpoints within the TME serve an important role in modulating host anti-tumor immunity. Several immune checkpoints within the TME have been reported, such as the B7 family, tumor necrosis factor, NK cells, extracellular nucleotides-related and phagocytosis checkpoints [22]. Subsequent discovery of additional immune checkpoint inhibitors and therapeutic predictors was of great practical value for HCC treatment. In the present study, we found that PD-1, CTLA-4, TIM3, TIGIT, CD8B, CD27, CD96, CD40LG and TNFRSF4 expression was upregulated, whilst ADORA2A, CD33, KLRC1, LAG3, PD-L1, Siglec 7 and Siglec 9 expression were downregulated in HCC. These results suggest that TRIM28 may be an important biomarker that can be used to evaluate the potential therapeutic effects of different immunotherapies on HCC.

H2AX is a variant of the H2A protein family and is a component of the histone octamer in nucleosomes [23, 24], which is widely applied as a tool for measuring DNA damage. H2AX has been reported to be associated with tumor size, vascular invasion, TNM stage and reduced survival after HCC transplantation [25]. Galanty et al. [26] revealed that H2AX served key roles in the ubiquitylation and SUMOylation of proteins regulating cellular responses [26]. H2AX recruits E3 ligase enzymes protein inhibitor of activated STAT (PIAS)4 and PIAS1 to promote responses to DNA double-strand breaks [27]. Furthermore, accumulating evidence shows the ubiquitination of H2AX serves vital roles in cancer and DNA damage [28–30]. In our study, H2AX expression was found to be significantly higher in HCC and was a downstream target of TRIM28. The interaction between TRIM28 and H2AX was likely to be consistent with that of ubiquitin-conjugating enzyme 2T or UBC13 [29, 30]. Further studies should be performed to deepen the understanding into how TRIM28 and H2AX interact during the ubiquitination process.

CDK4 is a key component that mainly regulates the G_1_-S transition of the cell cycle [31], apoptosis and HCC cell proliferation [32]. CDK4 is an important therapeutic target for cancer treatment, such that its inhibitors, including palbociclib, ponatinib and ribociclib [32–34], can block cell cycle progression, DNA damage response and immune modulation to suppress the cancer progression [35]. In addition, a previous study found that CDK4 is involved in the development of HCC [36]. CDK4 inhibition or treatment with the CDK4 inhibitor may represent a novel therapeutic strategy for HCC treatment, either alone or particularly in combination with sorafenib [33, 37]. We indicated in the present study that CDK4 is a downstream substrate of TRIM28 and H2AX in HCC, which suggests that TRIM28 and H2AX can serve as potential regulators of CDK4. This may assist in the refinement of clinical applications of CDK4 inhibitors for patients with HCC.

Clinical prediction models, including prognostic and diagnostic models, serve critical roles in predicting disease progression and survival. In the current study, we showed that higher expression levels of TRIM28, H2AX and CDK4 were associated with reduced overall survival, especially when all three proteins are simultaneously expressed at high levels. As shown in the data from our validation cohort, the ROC diagnostic value of TRIM28, H2AX and CDK4 was found to be 0.921, 0.795 and 0.890, respectively; whilst the area under the ROC curve of all three genes together is 0.933. These findings indicate that their combination can increase the diagnostic efficiency of HCC.

## CONCLUSIONS

In summary, TRIM28 expression was found to be upregulated in HCC, possibly due to the hypomethylation of cg05678175. In addition, TRIM28 expression was found to be associated with the immune state and clinical outcomes of patients with HCC, indicating its role in the development of HCC. Downstream, H2AX and CDK4 were found to be functional targets of TRIM28, whereby a nomogram was established. The TRIM28/H2AX/CDK4 combination was able to accurately predict disease progression and survival outcomes of patients with HCC. These data highlighted novel diagnostic and therapeutic strategies for the clinical management of HCCs.

## METHODS

### Gene expression analysis

TRIM expression data were obtained using GSE36411, GSE39791, GSE45267, GSE69715, and GSE87630, a computational method that is used to determine whether a previously defined set of genes show statistically significant differences in expression levels [38]. The different TRIM3, TRIM6 and TRIM28 expression datasets were available from TCGA data portal (http://www.cancer.gov/tcga, v30.0; September 23, 2021), GEPIA2 web server (http://gepia2.cancer-pku.cn/#analysis) and Oncomine gene expression array datasets. Additionally, we obtained violin plots showing TRIM3, TRIM6 and TRIM28 expression among the different pathological stages (stages I–IV) of all TCGA tumors using the “Pathological Stage Plot” module of GEPIA2.

### DNA methylation analysis

We explored the association between TRIM28 and their methylation state using the MethSurv tool (https://biit.cs.ut.ee/methsurv/). Spearman correlation coefficients were applied to estimate the correlation between TRIM28 expression and their gene methylation levels in TCGA-HCC.

### Immune checkpoints and infiltration analysis

Immune checkpoint data were derived from PubMed. Kaplan-Meier survival analysis was performed using the selected gene expression dataset from the HCC patient samples using Kaplan-Meier plotter (https://www.kmplot.com). The significance of co-occurrence or mutual exclusivity was calculated in cBioPortal web (https://www.cbioportal.org/). *Q* < 0.05 was considered to indicate a statistically significant result. To calculate the correlation of the expression levels of TRIM28 with tumor-infiltrating immune cells, Tumor Immune Estimation Resource (TIMER 2.0, https://cistrome.shinyapps.io/timer/) was used. We calculated the association between TRIM28 expression and a variety of tumor-infiltrating immune cell types, including CD8+ T cells, CD4+ T cells, B cells, macrophages, neutrophils and myeloid dendritic cells. In addition, the association between cumulative survival and TRIM28 copy numbers in HCC was also calculated in both TIMER 2.0 [39] and TCGA [40, 41].

### Gene enrichment analysis

We searched the cBioPortal website by using protein name ‘TRIM28’ and ‘*Homo sapiens*’ as the search terms. Parameters (evidence; experiments; low confidence 0.150 and ≤50 interactors) were set to obtain the potential TRIM28-binding proteins. In addition, GEPIA2 was used to obtain the top 50 genes associated with TRIM28 or H2AX based on the TCGA tumor and normal tissue datasets. A single-gene co-expression heat map was then made to show the differentially expressed genes (cBioPortal and GEPIA2) in the low- and high-expression tissue groups. A circos plot was also produced to show the Spearman correlation between TRIM28 expression and other mRNAs in HCC.

### Survival analysis

Survival plots analyzing TRIM28 expression in HCC were obtained using the ‘Survival Analysis’ module in TCGA. We then used the ‘Survival Map’ module of The Kaplan-Meier Plotter [42] to obtain the association between overall survival (OS), relapse-free survival (RFS), progression-free survival (PFS) or disease-specific survival DSS and TRIM28 or H2AX expression. Log-rank test was used to assess statistical significance.

### Tissue microarrays

Tissue microarrays (cat. no. HLivH180Su16) were purchased from Shanghai Outdo Biotech Co., Ltd., to detect the expression of TRIM28, H2AX and CDK4 gene.

### Immunohistochemical analysis (IHC)

Briefly, the microarrays were dewaxed in xylene and rehydrated in a descending ethanol gradient and PBS. Endogenous peroxidase was inactivated using 3% H_2_O_2_ and antigen retrieval was performed using the Tris-EDTA antigen retrieval buffer for TRIM28, H2AX and CDK4 staining. Sections were blocked with 10% normal goat serum at room temperature for 15 min and incubated with primary antibodies against TRIM28 (Santa Cruz Biotechnology, Inc.), H2AX (ab124781, Abcam) or CDK4 (ab108357, Abcam) at 4°C overnight. The sections were then incubated with corresponding secondary antibodies conjugated with horseradish peroxidase at room temperature for 30 min. The staining was visualized using a DAB kit (Beijing Zhongshan Golden Bridge Biotechnology Co., Ltd.; OriGene Technologies, Inc.). All sections were examined and scored independently by two investigators in a double-blinded manner. Staining and scoring were performed as previously described [43].

### Statistical analysis

The expression level of TRIM28 in patients with HCC was evaluated using scatter/box/stage plots GraphPad Prism 7 Software. A Mann-Whitney *U* test and Wilcoxon signed-rank sum test were used to compare the difference between two groups of data, while a Kruskal-Walli’s test was utilized to evaluate the difference among ≥ three groups. Further exploration was analyzed by post-hoc Bonferroni test or adjusted Bonferroni correction for multiple comparisons. A time-dependent ROC curve was used to calculate the diagnostic value of TRIM28, H2AX or CDK4 gene expression levels. Categorical variables are expressed in the form of quantity (percentage), and a χ^2^ test or Fisher’s exact test were used for comparison. Cox proportional-hazards model was used for univariate and multivariate survival analyses. Volcano plots were plotted using the Limma statistical package (version 3.52.2). Nomograms were constructed using the R package (Version 3.6.3) and calibration plots were generated to evaluate the performance of the nomogram. *P* < 0.05 was considered to indicate a statistically different difference.

### Limitation

In the present study, we have determined the role of TRIM28/H2AX/CDK4 associated with immune status in HCC. And the expression and prognosis of TRIM28, H2AX and CDK4 were validated further in an independent cohort of patients with HCC via IHC. However, there is still a lack of *in vivo* study for the expression of these three genes and the interaction between TRIM28, H2AX and CDK4.

### Availability of data and materials

The data and material can be found from correspondence author.

## Supplementary Materials

Supplementary Figures

Supplementary Tables

## References

[1] Sung H, Ferlay J, Siegel RL, Laversanne M, Soerjomataram I, Jemal A, Bray F. Global Cancer Statistics 2020: GLOBOCAN Estimates of Incidence and Mortality Worldwide for 36 Cancers in 185 Countries. CA Cancer J Clin. 2021; 71:209–49. 10.3322/caac.21660 33538338

[2] Zhang Y, Liu Y, Duan J, Yan H, Zhang J, Zhang H, Fan Q, Luo F, Yan G, Qiao K, Liu J. Hippocalcin-like 1 suppresses hepatocellular carcinoma progression by promoting p21(Waf/Cip1) stabilization by activating the ERK1/2-MAPK pathway. Hepatology. 2016; 63:880–97. 10.1002/hep.28395 26659654

[3] Wang W, Wei C. Advances in the early diagnosis of hepatocellular carcinoma. Genes Dis. 2020; 7:308–19. 10.1016/j.gendis.2020.01.014 32884985 PMC7452544

[4] Zhang H, Han W. Protein Post-translational Modifications in Head and Neck Cancer. Front Oncol. 2020; 10:571944. 10.3892/or.2017.5972 33117703 PMC7561398

[5] Hatakeyama S. TRIM Family Proteins: Roles in Autophagy, Immunity, and Carcinogenesis. Trends Biochem Sci. 2017; 42:297–311. 10.1016/j.tibs.2017.01.002 28118948

[6] Tiwari I, Yoon MH, Park BJ, Jang KL. Hepatitis C virus core protein induces epithelial-mesenchymal transition in human hepatocytes by upregulating E12/E47 levels. Cancer Lett. 2015; 362:131–8. 10.1016/j.canlet.2015.03.032 25817725

[7] Popovic D, Vucic D, Dikic I. Ubiquitination in disease pathogenesis and treatment. Nat Med. 2014; 20:1242–53. 10.1038/nm.3739 25375928

[8] Reymond A, Meroni G, Fantozzi A, Merla G, Cairo S, Luzi L, Riganelli D, Zanaria E, Messali S, Cainarca S, Guffanti A, Minucci S, Pelicci PG, Ballabio A. The tripartite motif family identifies cell compartments. EMBO J. 2001; 20:2140–51. 10.1093/emboj/20.9.2140 11331580 PMC125245

[9] Venuto S, Merla G. E3 Ubiquitin Ligase TRIM Proteins, Cell Cycle and Mitosis. Cells. 2019; 8:510. 10.3390/cells8050510 31137886 PMC6562728

[10] Napolitano LM, Meroni G. TRIM family: Pleiotropy and diversification through homomultimer and heteromultimer formation. IUBMB Life. 2012; 64:64–71. 10.1002/iub.580 22131136

[11] Watanabe M, Hatakeyama S. TRIM proteins and diseases. J Biochem. 2017; 161:135–44. 10.1093/jb/mvw087 28069866

[12] Guo P, Ma X, Zhao W, Huai W, Li T, Qiu Y, Zhang Y, Han L. TRIM31 is upregulated in hepatocellular carcinoma and promotes disease progression by inducing ubiquitination of TSC1-TSC2 complex. Oncogene. 2018; 37:478–88. 10.1038/onc.2017.349 28967907

[13] Zhang Y, Tao R, Wu SS, Xu CC, Wang JL, Chen J, Yu YS, Tang ZH, Chen XH, Zang GQ. TRIM52 up-regulation in hepatocellular carcinoma cells promotes proliferation, migration and invasion through the ubiquitination of PPM1A. J Exp Clin Cancer Res. 2018; 37:116. 10.1186/s13046-018-0780-9 29898761 PMC6001170

[14] Wang Y, Jiang J, Li Q, Ma H, Xu Z, Gao Y. KAP1 is overexpressed in hepatocellular carcinoma and its clinical significance. Int J Clin Oncol. 2016; 21:927–33. 10.1007/s10147-016-0979-8 27095111

[15] Li L, Dong L, Qu X, Jin S, Lv X, Tan G. Tripartite motif 16 inhibits hepatocellular carcinoma cell migration and invasion. Int J Oncol. 2016; 48:1639–49. 10.3892/ijo.2016.3398. Retraction in: Int J Oncol. 2023; 62:25. 10.3892/ijo.2016.3398 26892350

[16] Liang Q, Deng H, Li X, Wu X, Tang Q, Chang TH, Peng H, Rauscher FJ 3rd, Ozato K, Zhu F. Tripartite motif-containing protein 28 is a small ubiquitin-related modifier E3 ligase and negative regulator of IFN regulatory factor 7. J Immunol. 2011; 187:4754–63. 10.4049/jimmunol.1101704 21940674 PMC3197880

[17] Jin X, Pan Y, Wang L, Zhang L, Ravichandran R, Potts PR, Jiang J, Wu H, Huang H. MAGE-TRIM28 complex promotes the Warburg effect and hepatocellular carcinoma progression by targeting FBP1 for degradation. Oncogenesis. 2017; 6:e312. 10.1038/oncsis.2017.21 28394358 PMC5520498

[18] Zhang RY, Liu ZK, Wei D, Yong YL, Lin P, Li H, Liu M, Zheng NS, Liu K, Hu CX, Yang XZ, Chen ZN, Bian H. UBE2S interacting with TRIM28 in the nucleus accelerates cell cycle by ubiquitination of p27 to promote hepatocellular carcinoma development. Signal Transduct Target Ther. 2021; 6:64. 10.1038/s41392-020-00432-z 33589597 PMC7884418

[19] Li Z, Lu X, Liu Y, Zhao J, Ma S, Yin H, Huang S, Zhao Y, He X. Gain of LINC00624 Enhances Liver Cancer Progression by Disrupting the Histone Deacetylase 6/Tripartite Motif Containing 28/Zinc Finger Protein 354C Corepressor Complex. Hepatology. 2021; 73:1764–82. 10.1002/hep.31530 32869873

[20] Herquel B, Ouararhni K, Khetchoumian K, Ignat M, Teletin M, Mark M, Béchade G, Van Dorsselaer A, Sanglier-Cianférani S, Hamiche A, Cammas F, Davidson I, Losson R. Transcription cofactors TRIM24, TRIM28, and TRIM33 associate to form regulatory complexes that suppress murine hepatocellular carcinoma. Proc Natl Acad Sci U S A. 2011; 108:8212–7. 10.1073/pnas.1101544108 21531907 PMC3100982

[21] Farkona S, Diamandis EP, Blasutig IM. Cancer immunotherapy: the beginning of the end of cancer? BMC Med. 2016; 14:73. 10.1186/s12916-016-0623-5 27151159 PMC4858828

[22] Wei G, Zhang H, Zhao H, Wang J, Wu N, Li L, Wu J, Zhang D. Emerging immune checkpoints in the tumor microenvironment: Implications for cancer immunotherapy. Cancer Lett. 2021; 511:68–76. 10.1016/j.canlet.2021.04.021 33957184

[23] Ausió J, Abbott DW. The many tales of a tail: carboxyl-terminal tail heterogeneity specializes histone H2A variants for defined chromatin function. Biochemistry. 2002; 41:5945–9. 10.1021/bi020059d 11993987

[24] Kuo LJ, Yang LX. Gamma-H2AX - a novel biomarker for DNA double-strand breaks. In Vivo. 2008; 22:305–9. 18610740

[25] Xiao H, Tong R, Ding C, Lv Z, Du C, Peng C, Cheng S, Xie H, Zhou L, Wu J, Zheng S. γ-H2AX promotes hepatocellular carcinoma angiogenesis via EGFR/HIF-1α/VEGF pathways under hypoxic condition. Oncotarget. 2015; 6:2180–92. 10.18632/oncotarget.2942 25537504 PMC4385844

[26] Galanty Y, Belotserkovskaya R, Coates J, Jackson SP. RNF4, a SUMO-targeted ubiquitin E3 ligase, promotes DNA double-strand break repair. Genes Dev. 2012; 26:1179–95. 10.1101/gad.188284.112 22661229 PMC3371407

[27] Galanty Y, Belotserkovskaya R, Coates J, Polo S, Miller KM, Jackson SP. Mammalian SUMO E3-ligases PIAS1 and PIAS4 promote responses to DNA double-strand breaks. Nature. 2009; 462:935–9. 10.1038/nature08657 20016603 PMC2904806

[28] Rezaeian AH, Li CF, Wu CY, Zhang X, Delacerda J, You MJ, Han F, Cai Z, Jeong YS, Jin G, Phan L, Chou PC, Lee MH, et al. A hypoxia-responsive TRAF6-ATM-H2AX signalling axis promotes HIF1α activation, tumorigenesis and metastasis. Nat Cell Biol. 2017; 19:38–51. 10.1038/ncb3445 27918549 PMC5441459

[29] Ikura T, Tashiro S, Kakino A, Shima H, Jacob N, Amunugama R, Yoder K, Izumi S, Kuraoka I, Tanaka K, Kimura H, Ikura M, Nishikubo S, et al. DNA damage-dependent acetylation and ubiquitination of H2AX enhances chromatin dynamics. Mol Cell Biol. 2007; 27:7028–40. 10.1128/MCB.00579-07 17709392 PMC2168918

[30] Sun J, Zhu Z, Li W, Shen M, Cao C, Sun Q, Guo Z, Liu L, Wu D. UBE2T-regulated H2AX monoubiquitination induces hepatocellular carcinoma radioresistance by facilitating CHK1 activation. J Exp Clin Cancer Res. 2020; 39:222. 10.1186/s13046-020-01734-4 33087136 PMC7576867

[31] Pandey K, An HJ, Kim SK, Lee SA, Kim S, Lim SM, Kim GM, Sohn J, Moon YW. Molecular mechanisms of resistance to CDK4/6 inhibitors in breast cancer: A review. Int J Cancer. 2019; 145:1179–88. 10.1002/ijc.32020 30478914 PMC6767051

[32] Liu C, Mu X, Wang X, Zhang C, Zhang L, Yu B, Sun G. Ponatinib Inhibits Proliferation and Induces Apoptosis of Liver Cancer Cells, but Its Efficacy Is Compromised by Its Activation on PDK1/Akt/mTOR Signaling. Molecules. 2019; 24:1363. 10.3390/molecules24071363 30959969 PMC6480565

[33] Bollard J, Miguela V, Ruiz de Galarreta M, Venkatesh A, Bian CB, Roberto MP, Tovar V, Sia D, Molina-Sánchez P, Nguyen CB, Nakagawa S, Llovet JM, Hoshida Y, Lujambio A. Palbociclib (PD-0332991), a selective CDK4/6 inhibitor, restricts tumour growth in preclinical models of hepatocellular carcinoma. Gut. 2017; 66:1286–96. 10.1136/gutjnl-2016-312268 27849562 PMC5512174

[34] Prawira A, Le TBU, Vu TC, Huynh H. Ribociclib enhances infigratinib-induced cancer cell differentiation and delays resistance in FGFR-driven hepatocellular carcinoma. Liver Int. 2021; 41:608–20. 10.1111/liv.14728 33179425 PMC7894323

[35] Teo ZL, Versaci S, Dushyanthen S, Caramia F, Savas P, Mintoff CP, Zethoven M, Virassamy B, Luen SJ, McArthur GA, Phillips WA, Darcy PK, Loi S. Combined CDK4/6 and PI3Kα Inhibition Is Synergistic and Immunogenic in Triple-Negative Breast Cancer. Cancer Res. 2017; 77:6340–52. 10.1158/0008-5472.CAN-17-2210 28947417

[36] Wang H, Liao P, Zeng SX, Lu H. Co-targeting p53-R249S and CDK4 synergistically suppresses survival of hepatocellular carcinoma cells. Cancer Biol Ther. 2020; 21:269–77. 10.1080/15384047.2019.1685289 31747859 PMC7012101

[37] Jo H, Park Y, Kim T, Kim J, Lee JS, Kim SY, Chung JI, Ko HY, Pyun JC, Kim KS, Lee M, Yun M. Modulation of SIRT3 expression through CDK4/6 enhances the anti-cancer effect of sorafenib in hepatocellular carcinoma cells. BMC Cancer. 2020; 20:332. 10.1186/s12885-020-06822-4 32306906 PMC7168998

[38] Subramanian A, Tamayo P, Mootha VK, Mukherjee S, Ebert BL, Gillette MA, Paulovich A, Pomeroy SL, Golub TR, Lander ES, Mesirov JP. Gene set enrichment analysis: a knowledge-based approach for interpreting genome-wide expression profiles. Proc Natl Acad Sci U S A. 2005; 102:15545–50. 10.1073/pnas.0506580102 16199517 PMC1239896

[39] Li T, Fu J, Zeng Z, Cohen D, Li J, Chen Q, Li B, Liu XS. TIMER2.0 for analysis of tumor-infiltrating immune cells. Nucleic Acids Res. 2020; 48:W509–14. 10.1093/nar/gkaa407 32442275 PMC7319575

[40] Hänzelmann S, Castelo R, Guinney J. GSVA: gene set variation analysis for microarray and RNA-seq data. BMC Bioinformatics. 2013; 14:7. 10.1186/1471-2105-14-7 23323831 PMC3618321

[41] Bindea G, Mlecnik B, Tosolini M, Kirilovsky A, Waldner M, Obenauf AC, Angell H, Fredriksen T, Lafontaine L, Berger A, Bruneval P, Fridman WH, Becker C, et al. Spatiotemporal dynamics of intratumoral immune cells reveal the immune landscape in human cancer. Immunity. 2013; 39:782–95. 10.1016/j.immuni.2013.10.003 24138885

[42] Menyhárt O, Nagy Á, Győrffy B. Determining consistent prognostic biomarkers of overall survival and vascular invasion in hepatocellular carcinoma. R Soc Open Sci. 2018; 5:181006. 10.1098/rsos.181006 30662724 PMC6304123

[43] Lu G, Tian S, Sun Y, Dong J, Wang N, Zeng J, Nie Y, Wu K, Han Y, Feng B, Shang Y. NEK9, a novel effector of IL-6/STAT3, regulates metastasis of gastric cancer by targeting ARHGEF2 phosphorylation. Theranostics. 2021; 11:2460–74. 10.7150/thno.53169 33500736 PMC7797683

