# Supplementary material for: Integrated analysis and validation of the TRIM28-H2AX-CDK4 diagnostic model assists to predict the progression of HCC
Source: Aging (Albany NY). 2023 Oct 20;15(24):14617–50. doi: 10.18632/aging.205137 (PMC10781449; doi:10.18632/aging.205137)
Supplement: Supplementary Figures [file aging-15-205137-s001.pdf]

SUPPLEMENTARY FIGURES

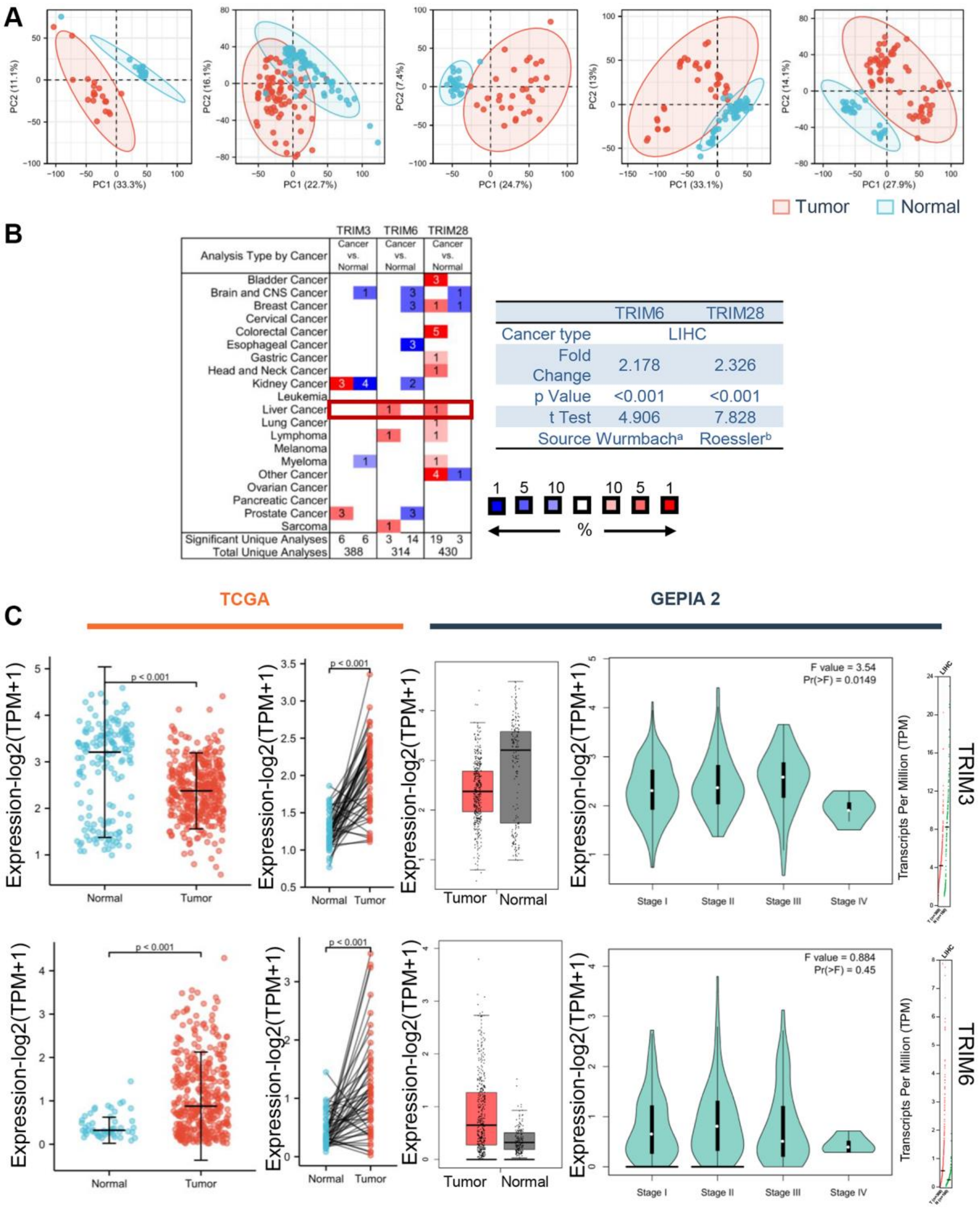

Supplementary Figure 1. The expression levels of TRIMs protein and clinical features in HCC. (**A**) PCA plots showed differential gene expression in HCC and adjacent nontumor tissues from GSEA database (red: HCC tissues, blue: adjacent nontumor tissues). (**B**) TRIM6 and TRIM28 were showed to be increased in HCC from Oncomine data (red: overexpression, blue: down expression). (**C**) We analyzed the expression level of TRIM28 total protein between normal tissue and HCC tissue from TCGA, $p < 0.001$ (left). Based on the GEPIA 2 data, the TRIM28 expression levels was analyzed by main pathological stages (stage I, stage II, stage III, and stage IV) (right). Log2 (TPM + 1) was applied for log-scale.

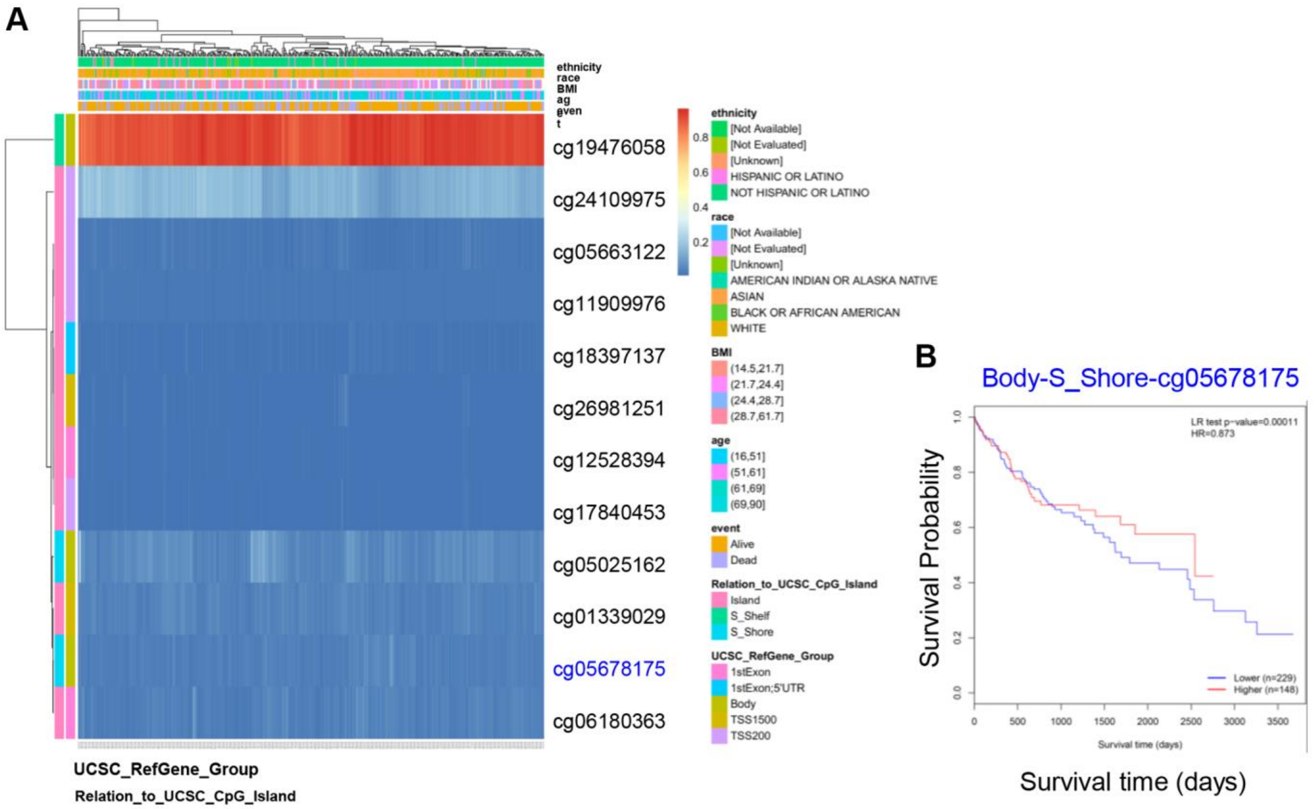

Supplementary Figure 2. DNA sequences and methylation sites of TRIM28 were identified using MethSurv in HCC. (**A**) The methylation site of TRIM28 DNA sequence association with gene expression was visualized using MEXPRESS. The expression of TRIM28 is illustrated by the blue line in the center of the plot. Pearson's correlation coefficients and *p* values for methylation sites and query gene expression are shown on the right side, relationship between TRIM28 expression and methylation site cg05678175 by Kaplan-Meier Plotter (**B**).

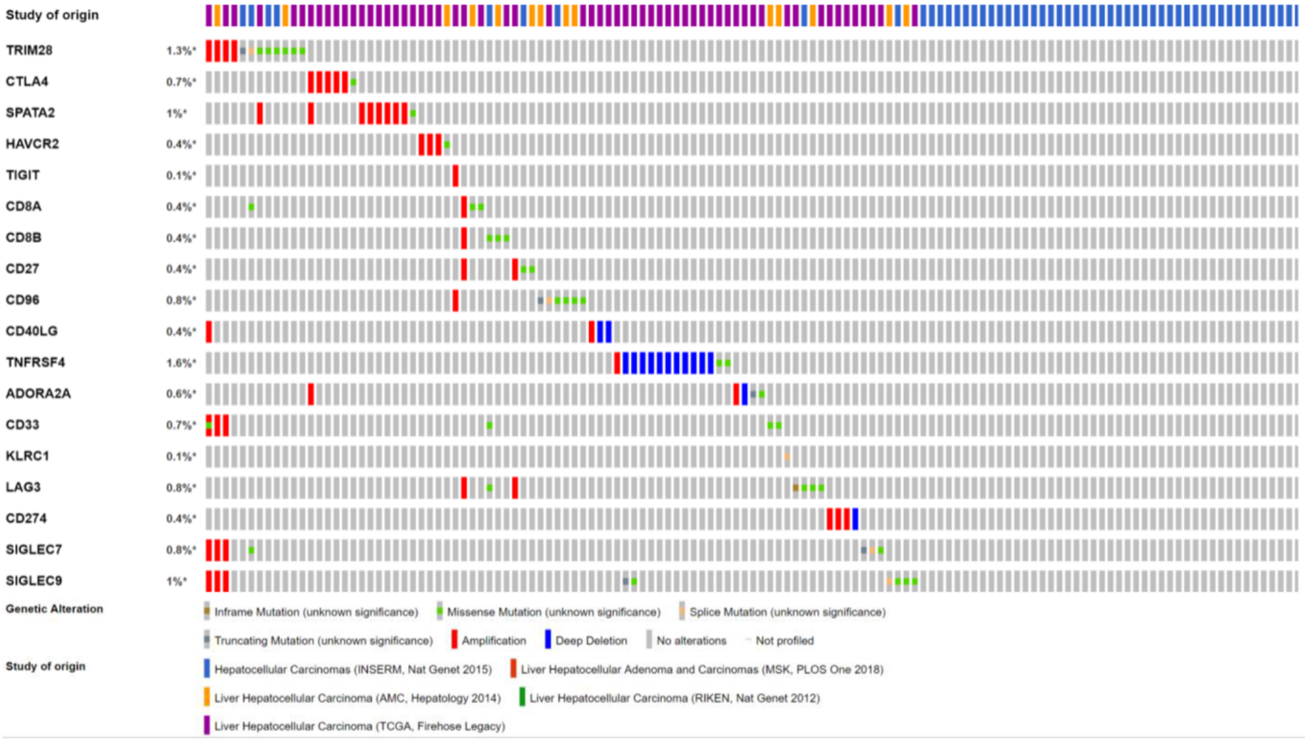

Supplementary Figure 3. Correlations between TRIM28 and immune checkpoints in HCC. Landscape of TRIM28 expression level and immune checkpoint alteration in HCC. Compact visualization of cases with multiple genetic alterations of TRIM28 and immune checkpoints were individually shown by cBioPortal as indicated, including fusion, amplification, deep deletion, truncating mutation, and missense mutation.

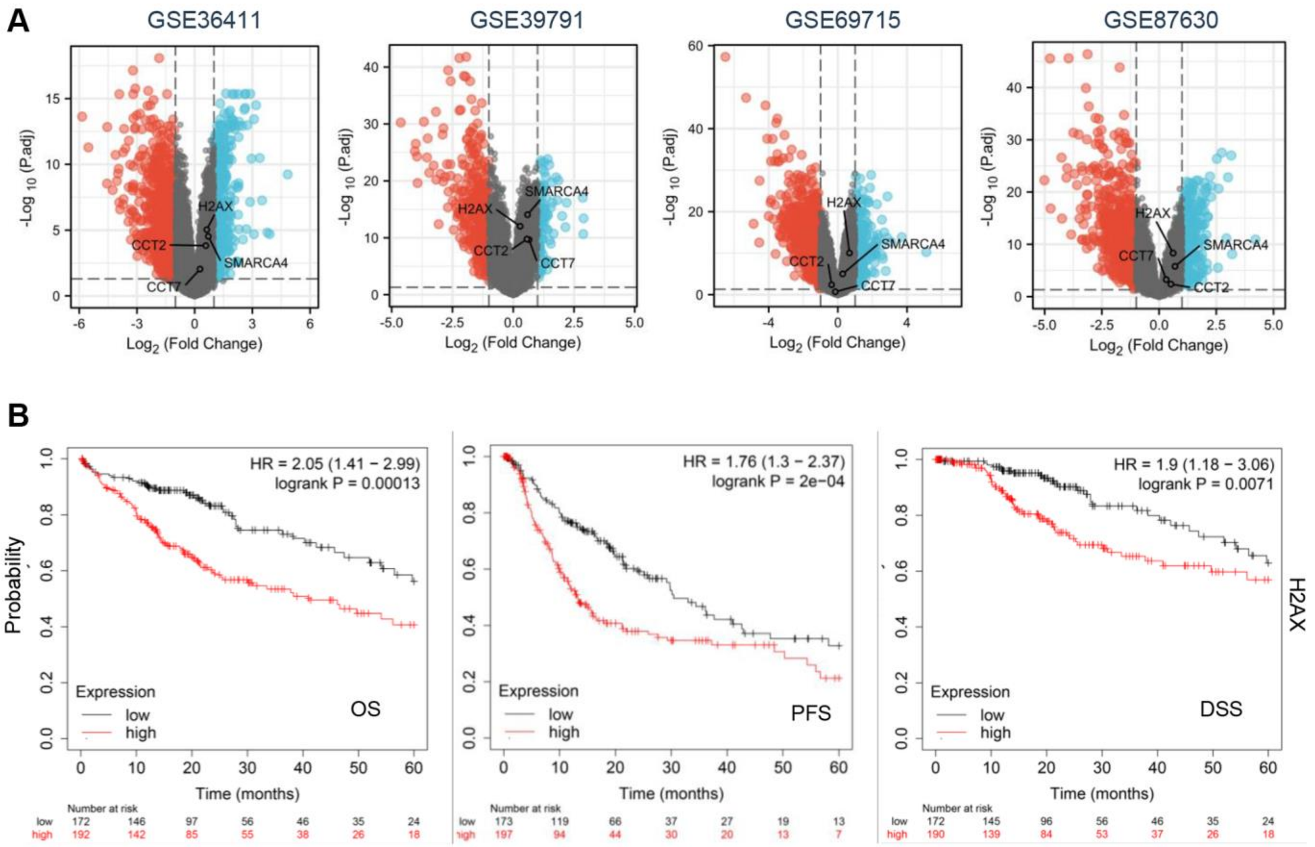

Supplementary Figure 4. The positive correlation of TRIM28 and H2AX and their prognostic values in HCC. (**A**) An overview of CCT2, CCT7, H2AX and SMARCA4 expression in HCC from GSE36411, GSE39791, GSE69715, GSE87630 from TCGA data (blue: overexpression, red: down expression, grey: no significant). (**B**) Survival analysis using the Kaplan-Meier plotter tool OF H2AX expression and OS, PFS and DSS in HCC.

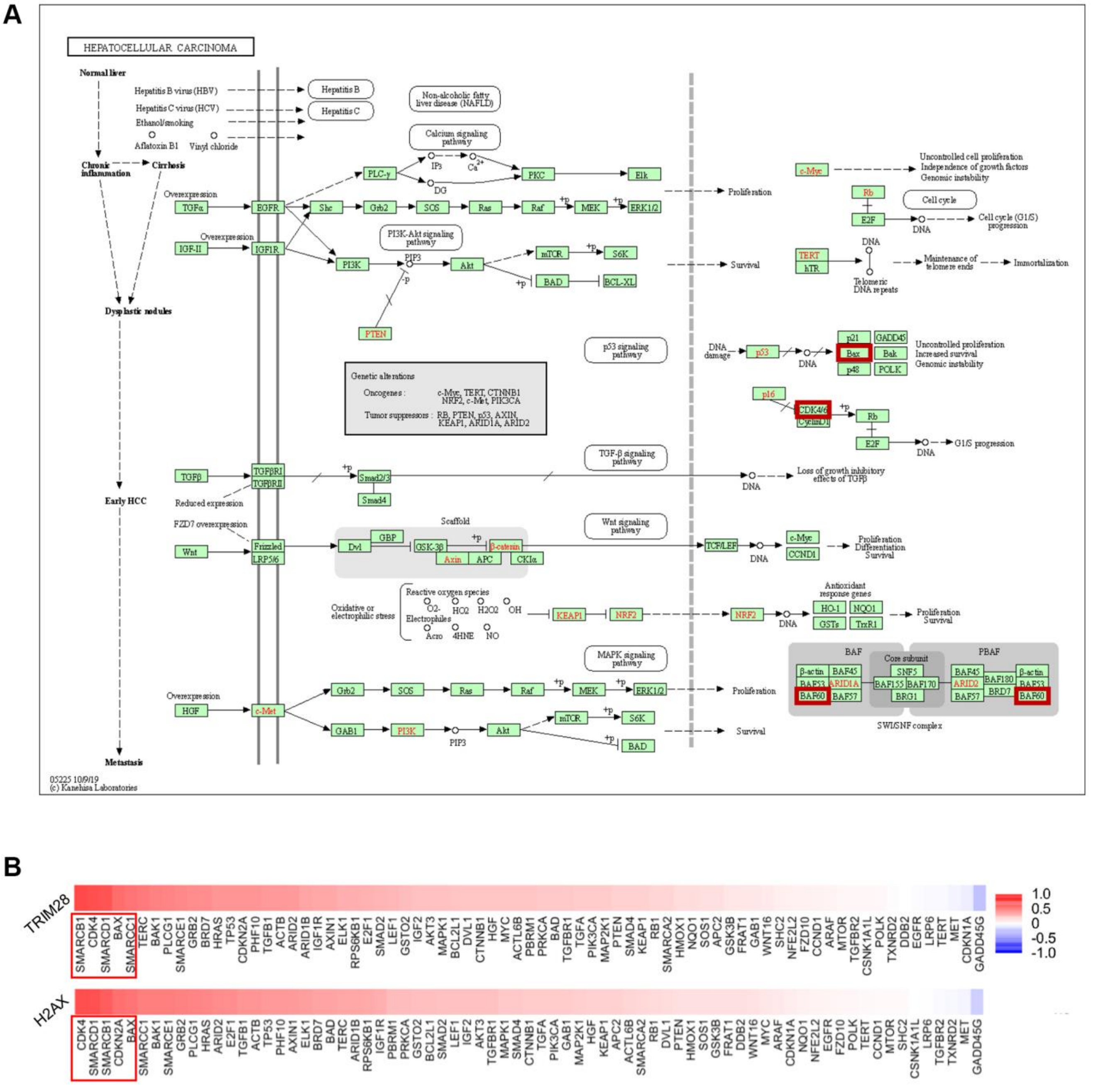

Supplementary Figure 5. CDK4 was the downstream gene of TRIM28 and H2AX. (**A**) A KEGG mapping 05225 suggested the protentional signalling pathways in HCC. (**B**) The top 50 genes with positive association with TRIM28 and H2AX interacting genes from KEGG signalling pathways in HCC.

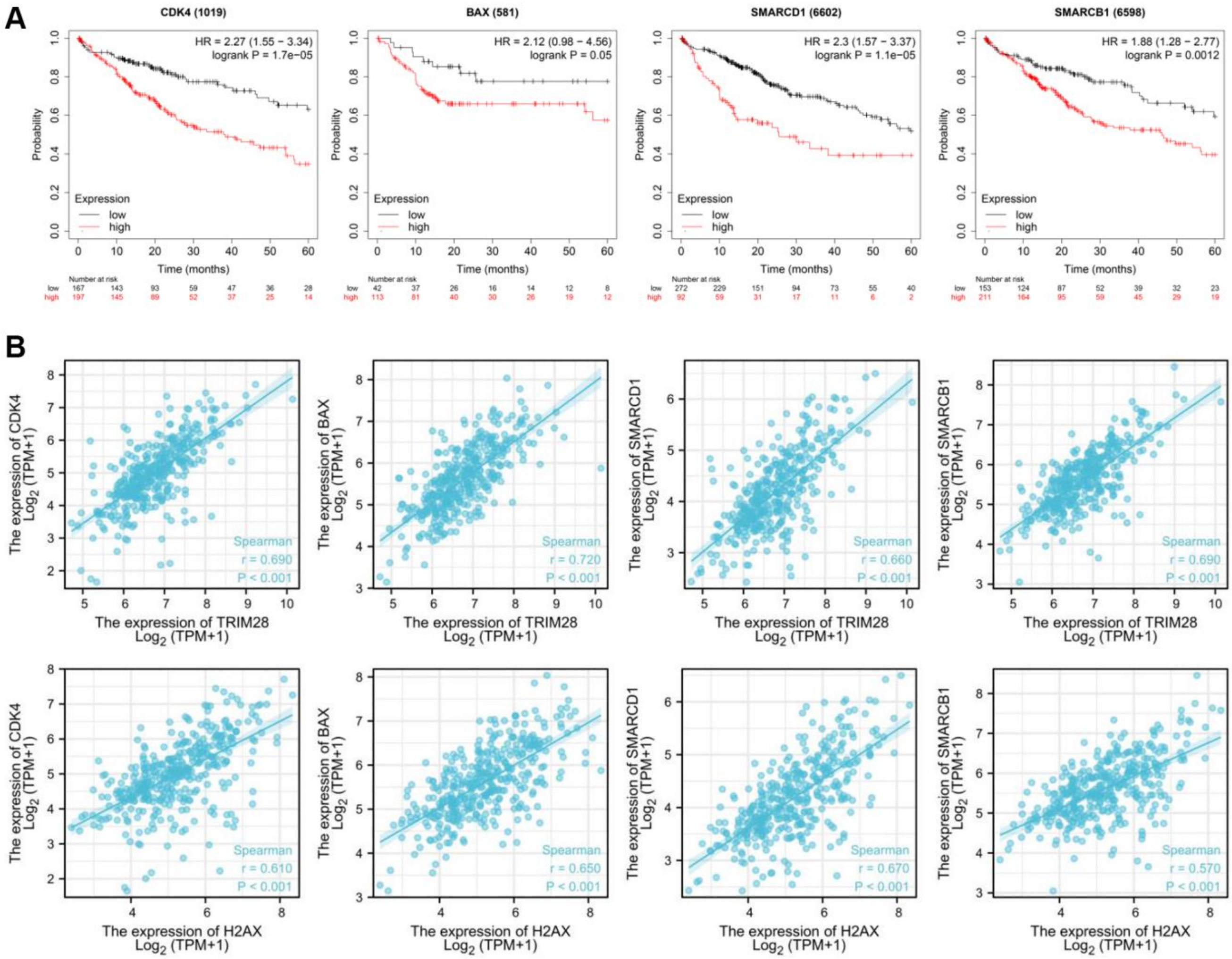

Supplementary Figure 6. Correlation between TRIM28 or H2AX and these candidate genes. (**A**) Overall survival analyzed determined that CDK4, BAX, SMARCD1 and SMARCB1 is involved in poor prognosis of patients with HCC patients from Kaplan-Meier Plotter. (**B**) A positively correlated between TRIM28 or H2AX expression with those four genes in HCC from TCGA data.

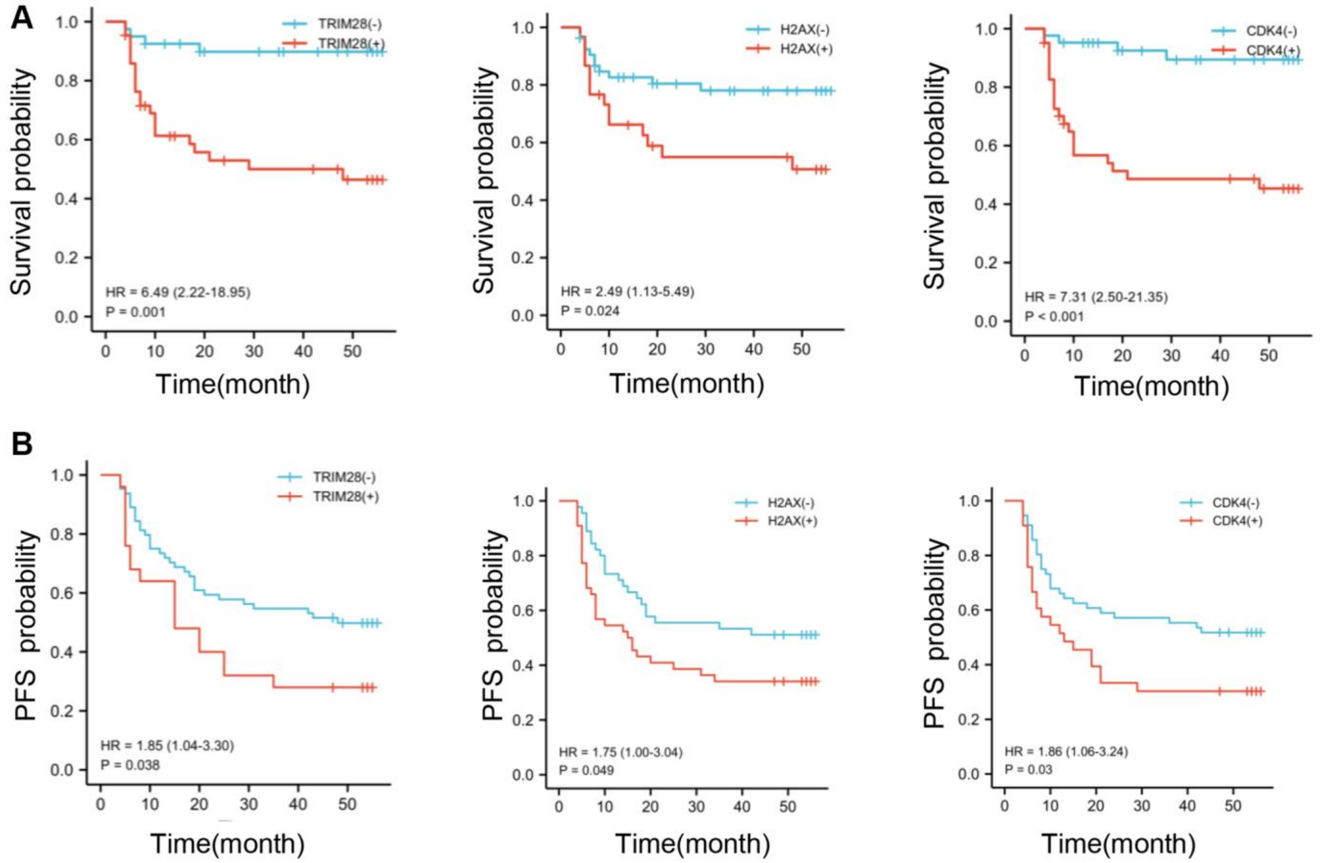

Supplementary Figure 7. Relationship between TRIM28 expression with overall survival (OS) and recurrence in 90 HCCs. (**A**) A Kaplan-Meier analysis of TRIM28, H2AX and CDK4 expression for OS from an independent cohort of HCC. (**B**) Analysis of TRIM28, H2AX and CDK4 expression for PFS from an independent cohort of HCC.
