# Supplementary material for: Integrated analysis and validation of the TRIM28-H2AX-CDK4 diagnostic model assists to predict the progression of HCC
Source: Aging (Albany NY). 2023 Oct 20;15(24):14617–50. doi: 10.18632/aging.205137 (PMC10781449; doi:10.18632/aging.205137)
Supplement: Supplementary Tables [file aging-15-205137-s002.pdf]

SUPPLEMENTARY TABLES

Supplementary Table 1. The reference information of CpGs.

Name	R (copy number)	HR	CI	*P* value	CHR	UCSC_RefGene_Group	Relation_to_UCSC_CpG_Island
cg05663122	−0.050	1.908	(1.221, 2.981)	0.005	19	TSS200	Island
cg05025162	0.127	1.673	(1.185, 2.362)	0.003	19	Body	S_Shore
cg26981251	−0.003	1.59	(1.118, 2.261)	0.010	19	TSS1500	Island
cg24109975	0.030	1.522	(1.071, 2.164)	0.019	19	TSS200	Island
cg12528394	−0.069	1.481	(1.046, 2.096)	0.027	19	1stExon	Island
cg01339029	0.090	1.373	(0.899, 2.097)	0.142	19	TSS1500	Island
cg11909976	−0.170	1.205	(0.777, 1.868)	0.405	19	TSS200	Island
cg19476058	−0.230	1.124	(0.745, 1.693)	0.578	19	Body	S_Shelf
cg17840453	−0.065	1.121	(0.782, 1.605)	0.535	19	TSS200	Island
cg18397137	−0.099	1.093	(0.77, 1.55)	0.619	19	1stExon;5′UTR	Island
cg06180363	0.113	0.829	(0.588, 1.17)	0.286	19	1stExon	Island
cg05678175	−0.205	0.677	(0.479, 0.959)	0.028	19	Body	S_Shore

Supplementary Table 2. Prognostic summary of immune checkpoints.

Checkpoint	Expression	OS	*p*-Value	HR (high)	RFS	*p*-Value	HR (high)	PFS	*p*-Value	HR (high)	DSS	*p*-Value	HR (high)
CTLA4	Up	Ns	0.201	0.79	Favor	0.018	0.65	Favor	0.04	0.71	Ns	0.309	0.78
PD1	Up	Ns	0.223	1.24	Unfavor	0.029	1.47	Unfavor	0.016	1.47	Ns	0.156	1.45
TIM3	Up	Ns	0.226	1.28	Favor	0.02	0.66	Favor	0.02	0.7	Ns	0.342	0.79
TIGIT	Up	Ns	0.067	0.71	Favor	0.001	0.58	Favor	0.002	0.6	Favor	0.044	0.62
CD8A	Ns	Favor	0.001	0.56	Favor	0.001	0.57	Favor	<0.001	0.57	Favor	0.001	0.48
CD8B	Up	Favor	0.006	0.6	Favor	0.001	0.57	Favor	0.001	0.58	Favor	0.008	0.52
CD27	Up	Favor	0.006	0.59	Favor	0.007	0.58	Favor	0.003	0.59	Favor	0.019	0.59
CD96	Up	Favor	0.001	0.56	Favor	<0.001	0.49	Favor	<0.001	0.51	Favor	0.001	0.45
CD40LG	Up	Favor	0.006	0.6	Favor	0.001	0.55	Favor	0.004	0.65	Favor	0.006	0.53
TNFRSF4	Up	Unfavor	<0.001	2.08	Unfavor	0.005	1.67	Unfavor	0.015	1.48	Unfavor	<0.001	2.59
ADORA2A	Down	Favor	0.006	0.59	Favor	0.009	0.65	Favor	0.035	0.73	Favor	0.025	0.59
CD33	Down	Favor	0.041	0.7	Favor	0.006	0.63	Favor	0.003	0.64	Favor	0.025	0.6
KLRC1	Down	Favor	0.008	0.61	Favor	0.001	0.55	Favor	0.008	0.65	Favor	0.034	0.61
LAG3	Down	Favor	0.022	0.6	Favor	0.016	0.67	Favor	0.007	0.66	Ns	0.162	0.68
PD-L1	Down	Ns	0.095	0.71	Ns	0.234	0.81	Ns	0.266	0.84	Ns	0.096	0.64
Siglec7	Down	Ns	0.146	0.77	Favor	0.003	0.61	Favor	0.001	0.61	Ns	0.078	0.66
Siglec9	Down	Ns	0.201	1.25	Favor	0.028	0.67	Favor	0.038	0.69	Ns	0.42	0.81

Supplementary Table 3. Mutual Exclusivity.

A	B	Neither	A Not B	B Not A	Both	Log2 odds ratio	*p*-Value	*q*-Value	Tendency	Significance
TRIM28	**SIGLEC7**	852	8	3	4	>3	<0.001	**<0.001**	Co-occurrence	**Yes**
TRIM28	**CD33**	852	9	3	3	>3	<0.001	**0.001**	Co-occurrence	**Yes**
TRIM28	**SIGLEC9**	849	9	6	3	>3	<0.001	**0.004**	Co-occurrence	**Yes**
TRIM28	CD8A	852	11	3	1	>3	0.054	0.346	Co-occurrence	No
TRIM28	CD40LG	852	11	3	1	>3	0.054	0.346	Co-occurrence	No
TRIM28	SPATA2	847	11	8	1	>3	0.118	0.697	Co-occurrence	No
TRIM28	TNFRSF4	841	12	14	0	<−3	0.822	0.999	Mutual exclusivity	No
TRIM28	CD96	848	12	7	0	<−3	0.907	0.999	Mutual exclusivity	No
TRIM28	LAG3	848	12	7	0	<−3	0.907	0.999	Mutual exclusivity	No
TRIM28	CTLA4	849	12	6	0	<−3	0.92	0.999	Mutual exclusivity	No
TRIM28	ADORA2A	850	12	5	0	<−3	0.933	0.999	Mutual exclusivity	No
TRIM28	HAVCR2	851	12	4	0	<−3	0.946	0.999	Mutual exclusivity	No

TRIM28	CD8B	851	12	4	0	<−3	0.946	0.999	Mutual exclusivity	No
TRIM28	CD27	851	12	4	0	<−3	0.946	0.999	Mutual exclusivity	No
TRIM28	CD274	851	12	4	0	<−3	0.946	0.999	Mutual exclusivity	No
TRIM28	TIGIT	854	12	1	0	<−3	0.986	0.999	Mutual exclusivity	No
TRIM28	KLRC1	854	12	1	0	<−3	0.986	0.999	Mutual exclusivity	No

Supplementary Table 4. Univariate and multivariate analysis of CDK4 associated with DSS of HCC.

Characteristics	Total (*N*)	Univariate analysis		Multivariate analysis	
		**Hazard ratio (95% CI)**	***P* value**	**Hazard ratio (95% CI)**	***P* value**
T stage (T2 & T3 & T4 vs. T1)	362	2.829 (1.747–4.582)	**<0.001**	0.284 (0.033–2.449)	0.252
Pathologic stage (Stage II & Stage III & Stage IV vs. Stage I)	341	2.909 (1.718–4.925)	**<0.001**	8.223 (0.921–73.410)	0.059
Age (>60 vs. ≤60)	365	0.846 (0.543–1.317)	0.458	1.013 (0.548–1.873)	0.968
BMI (>25 vs. ≤25)	329	0.826 (0.512–1.330)	0.431	1.414 (0.750–2.664)	0.284
Histologic grade (G3 & G4 vs. G1 & G2)	360	1.086 (0.683–1.728)	0.726	1.227 (0.638–2.358)	0.540
AFP (ng/ml) (>400 vs. ≤400)	275	0.867 (0.450–1.668)	0.668	0.426 (0.186–0.977)	**0.044**
CDK4 (High vs. Low)	365	2.346 (1.479–3.721)	**<0.001**	3.025 (1.516–6.034)	**0.002**

Supplementary Table 5. Univariate and multivariate analysis of CDK4 associated with OS of HCC.

Characteristics	Total (*N*)	Univariate analysis		Multivariate analysis	
		**Hazard ratio (95% CI)**	***P* value**	**Hazard ratio (95% CI)**	***P* value**
T stage (T2 & T3 & T4 vs. T1)	370	2.126 (1.481–3.052)	**<0.001**	0.500 (0.063–3.965)	0.512
Pathologic stage (Stage II & Stage III & Stage IV vs. Stage I)	349	2.090 (1.429–3.055)	**<0.001**	3.606 (0.446–29.136)	0.229
Age (>60 vs. ≤60)	373	1.205 (0.850–1.708)	0.295	1.592 (0.979–2.589)	0.061
BMI (>25 vs. ≤25)	336	0.798 (0.550–1.158)	0.235	1.129 (0.695–1.833)	0.624
Histologic grade (G3 & G4 vs. G1 & G2)	368	1.091 (0.761–1.564)	0.636	1.487 (0.900–2.457)	0.122
AFP (ng/ml) (>400 vs. ≤400)	279	1.075 (0.658–1.759)	0.772	0.729 (0.404–1.316)	0.294
CDK4 (High vs. Low)	373	1.921 (1.350–2.733)	**<0.001**	1.890 (1.121–3.187)	**0.017**

Supplementary Table 6. Univariate and multivariate analysis of CDK4 associated with PFS of HCC.

Characteristics	Total (*N*)	Univariate analysis		Multivariate analysis	
		**Hazard ratio (95% CI)**	***P* value**	**Hazard ratio (95% CI)**	***P* value**
T stage (T2 & T3 & T4 vs. T1)	370	2.360 (1.745–3.191)	**<0.001**	0.470 (0.061–3.605)	0.467
Pathologic stage (Stage II & Stage III & Stage IV vs. Stage I)	349	2.284 (1.670–3.122)	**<0.001**	4.121 (0.536–31.713)	0.174
Age (>60 vs. ≤60)	373	0.960 (0.718–1.284)	0.783	1.042 (0.725–1.498)	0.823
BMI (>25 vs. ≤25)	336	0.936 (0.689–1.272)	0.673	1.085 (0.748–1.574)	0.665
Histologic grade (G3 & G4 vs. G1 & G2)	368	1.152 (0.853–1.557)	0.355	1.006 (0.690–1.465)	0.976
AFP (ng/ml) (>400 vs. ≤400)	279	1.045 (0.698–1.563)	0.832	0.775 (0.496–1.211)	0.263
CDK4 (High vs. Low)	373	1.807 (1.348–2.424)	**<0.001**	1.868 (1.277–2.733)	**0.001**

Supplementary Table 7. Univariate and multivariate analysis of BAX associated with DSS of HCC.

Characteristics	Total (*N*)	Univariate analysis		Multivariate analysis	
		Hazard ratio (95% CI)	*P* value	Hazard ratio (95% CI)	*P* value
T stage (T2 & T3 & T4 vs. T1)	362	2.829 (1.747–4.582)	**<0.001**	0.213 (0.025–1.856)	0.162
Pathologic stage (Stage II & Stage III & Stage IV vs. Stage I)	341	2.909 (1.718–4.925)	**<0.001**	9.104 (0.996–83.209)	0.050
Age (>60 vs. ≤60)	365	0.846 (0.543–1.317)	0.458	1.013 (0.548–1.872)	0.968
BMI (>25 vs. ≤25)	329	0.826 (0.512–1.330)	0.431	1.366 (0.730–2.558)	0.329
Histologic grade (G3 & G4 vs. G1 & G2)	360	1.086 (0.683–1.728)	0.726	1.482 (0.782–2.809)	0.228
AFP (ng/ml) (>400 vs. ≤400)	275	0.867 (0.450–1.668)	0.668	0.661 (0.305–1.433)	0.294
BAX (High vs. Low)	365	1.378 (0.885–2.144)	0.156	1.185 (0.625–2.248)	0.604

Supplementary Table 8. Univariate and multivariate analysis of BAX associated with OS of HCC.

Characteristics	Total (*N*)	Univariate analysis		Multivariate analysis	
		Hazard ratio (95% CI)	*P* value	Hazard ratio (95% CI)	*P* value
T stage (T2 & T3 & T4 vs. T1)	370	2.126 (1.481–3.052)	**<0.001**	0.448 (0.056–3.568)	0.448
Pathologic stage (Stage II & Stage III & Stage IV vs. Stage I)	349	2.090 (1.429–3.055)	**<0.001**	3.566 (0.437–29.074)	0.235
Age (>60 vs. ≤60)	373	1.205 (0.850–1.708)	0.295	1.619 (0.997–2.631)	0.052
BMI (>25 vs. ≤25)	336	0.798 (0.550–1.158)	0.235	1.163 (0.717–1.887)	0.541
Histologic grade (G3 & G4 vs. G1 & G2)	368	1.091 (0.761–1.564)	0.636	1.612 (0.984–2.640)	0.058
AFP (ng/ml) (>400 vs. ≤400)	279	1.075 (0.658–1.759)	0.772	0.863 (0.490–1.519)	0.609
BAX (High vs. Low)	373	1.475 (1.043–2.085)	**0.028**	1.401 (0.855–2.297)	0.181

Supplementary Table 9. Univariate and multivariate analysis of BAX associated with PFS of HCC.

Characteristics	Total (*N*)	Univariate analysis		Multivariate analysis	
		Hazard ratio (95% CI)	*P* value	Hazard ratio (95% CI)	*P* value
T stage (T2 & T3 & T4 vs. T1)	370	2.360 (1.745–3.191)	**<0.001**	0.349 (0.045–2.694)	0.313
Pathologic stage (Stage II & Stage III & Stage IV vs. Stage I)	349	2.284 (1.670–3.122)	**<0.001**	5.215 (0.670–40.561)	0.115
Age (>60 vs. ≤60)	373	0.960 (0.718–1.284)	0.783	1.005 (0.700–1.443)	0.979
BMI (>25 vs. ≤25)	336	0.936 (0.689–1.272)	0.673	1.075 (0.742–1.558)	0.701
Histologic grade (G3 & G4 vs. G1 & G2)	368	1.152 (0.853–1.557)	0.355	1.140 (0.781–1.664)	0.496
AFP (ng/ml) (>400 vs. ≤400)	279	1.045 (0.698–1.563)	0.832	0.940 (0.605–1.459)	0.781
BAX (High vs. Low)	373	1.295 (0.969–1.732)	0.081	0.950 (0.656–1.377)	0.788

Supplementary Table 10. Univariate and multivariate analysis of SMARCD1 associated with DSS of HCC.

Characteristics	Total (*N*)	Univariate analysis		Multivariate analysis	
		Hazard ratio (95% CI)	*P* value	Hazard ratio (95% CI)	*P* value
T stage (T2 & T3 & T4 vs. T1)	362	2.829 (1.747–4.582)	**<0.001**	0.275 (0.032–2.364)	0.239
Pathologic stage (Stage II & Stage III & Stage IV vs. Stage I)	341	2.909 (1.718–4.925)	**<0.001**	7.083 (0.788–63.667)	0.081
Age (>60 vs. ≤60)	365	0.846 (0.543–1.317)	0.458	1.006 (0.543–1.863)	0.985
BMI (>25 vs. ≤25)	329	0.826 (0.512–1.330)	0.431	1.380 (0.738–2.580)	0.313
Histologic grade (G3 & G4 vs. G1 & G2)	360	1.086 (0.683–1.728)	0.726	1.184 (0.613–2.289)	0.615
AFP (ng/ml) (>400 vs. ≤400)	275	0.867 (0.450–1.668)	0.668	0.522 (0.235–1.159)	0.110
SMARCD1 (High vs. Low)	365	2.050 (1.303–3.225)	**0.002**	2.400 (1.216–4.740)	**0.012**

Supplementary Table 11. Univariate and multivariate analysis of SMARCD1 associated with OS of HCC.

Characteristics	Total (*N*)	Univariate analysis		Multivariate analysis	
		Hazard ratio (95% CI)	*P* value	Hazard ratio (95% CI)	*P* value
T stage (T2 & T3 & T4 vs. T1)	370	2.126 (1.481–3.052)	**<0.001**	0.499 (0.063–3.959)	0.511
Pathologic stage (Stage II & Stage III & Stage IV vs. Stage I)	349	2.090 (1.429–3.055)	**<0.001**	3.241 (0.399–26.351)	0.271
Age (>60 vs. ≤60)	373	1.205 (0.850–1.708)	0.295	1.603 (0.984–2.613)	0.058
BMI (>25 vs. ≤25)	336	0.798 (0.550–1.158)	0.235	1.130 (0.698–1.829)	0.620
Histologic grade (G3 & G4 vs. G1 & G2)	368	1.091 (0.761–1.564)	0.636	1.450 (0.876–2.402)	0.149
AFP (ng/ml) (>400 vs. ≤400)	279	1.075 (0.658–1.759)	0.772	0.797 (0.450–1.411)	0.436
SMARCD1 (High vs. Low)	373	1.934 (1.360–2.750)	**<0.001**	1.818 (1.086–3.042)	**0.023**

Supplementary Table 12. Univariate and multivariate analysis of SMARCD1 associated with PFS of HCC.

Characteristics	Total (*N*)	Univariate analysis		Multivariate analysis	
		Hazard ratio (95% CI)	*P* value	Hazard ratio (95% CI)	*P* value
T stage (T2 & T3 & T4 vs. T1)	370	2.360 (1.745–3.191)	**<0.001**	0.398 (0.052–3.061)	0.376
Pathologic stage (Stage II & Stage III & Stage IV vs. Stage I)	349	2.284 (1.670–3.122)	**<0.001**	4.525 (0.585–35.001)	0.148
Age (>60 vs. ≤60)	373	0.960 (0.718–1.284)	0.783	0.997 (0.694–1.433)	0.989
BMI (>25 vs. ≤25)	336	0.936 (0.689–1.272)	0.673	1.083 (0.748–1.567)	0.674
Histologic grade (G3 & G4 vs. G1 & G2)	368	1.152 (0.853–1.557)	0.355	1.066 (0.730–1.555)	0.742
AFP (ng/ml) (>400 vs. ≤400)	279	1.045 (0.698–1.563)	0.832	0.871 (0.557–1.360)	0.542
SMARCD1 (High vs. Low)	373	1.471 (1.100–1.968)	**0.009**	1.299 (0.890–1.894)	0.175

Supplementary Table 13. Univariate and multivariate analysis of SMARCB1 associated with DSS of HCC.

Characteristics	Total (*N*)	Univariate analysis		Multivariate analysis	
		Hazard ratio (95% CI)	*P* value	Hazard ratio (95% CI)	*P* value
T stage (T2 & T3 & T4 vs. T1)	362	2.829 (1.747–4.582)	**<0.001**	0.203 (0.023–1.760)	0.148
Pathologic stage (Stage II & Stage III & Stage IV vs. Stage I)	341	2.909 (1.718–4.925)	**<0.001**	9.798 (1.088–88.236)	**0.042**
Age (>60 vs. ≤60)	365	0.846 (0.543–1.317)	0.458	0.998 (0.540–1.844)	0.994
BMI (>25 vs. ≤25)	329	0.826 (0.512–1.330)	0.431	1.355 (0.724–2.535)	0.343
Histologic grade (G3 & G4 vs. G1 & G2)	360	1.086 (0.683–1.728)	0.726	1.513 (0.797–2.869)	0.205
AFP (ng/ml) (>400 vs. ≤400)	275	0.867 (0.450–1.668)	0.668	0.673 (0.301–1.505)	0.335
SMARCB1 (High vs. Low)	365	1.671 (1.068–2.615)	**0.025**	1.053 (0.541–2.050)	0.880

Supplementary Table 14. Univariate and multivariate analysis of SMARCB1 associated with OS of HCC.

Characteristics	Total (*N*)	Univariate analysis		Multivariate analysis	
		Hazard ratio (95% CI)	*P* value	Hazard ratio (95% CI)	*P* value
T stage (T2 & T3 & T4 vs. T1)	370	2.126 (1.481–3.052)	**<0.001**	0.380 (0.048–3.034)	0.362
Pathologic stage (Stage II & Stage III & Stage IV vs. Stage I)	349	2.090 (1.429–3.055)	**<0.001**	4.264 (0.525–34.630)	0.175
Age (>60 vs. ≤60)	373	1.205 (0.850–1.708)	0.295	1.600 (0.984–2.601)	0.058
BMI (>25 vs. ≤25)	336	0.798 (0.550–1.158)	0.235	1.137 (0.702–1.842)	0.602
Histologic grade (G3 & G4 vs. G1 & G2)	368	1.091 (0.761–1.564)	0.636	1.722 (1.042–2.845)	**0.034**
AFP (ng/ml) (>400 vs. ≤400)	279	1.075 (0.658–1.759)	0.772	0.946 (0.523–1.710)	0.854
SMARCB1 (High vs. Low)	373	1.489 (1.053–2.107)	**0.024**	0.945 (0.561–1.591)	0.831

Supplementary Table 15. Univariate and multivariate analysis of SMARCB1 associated with PFI of HCC.

Characteristics	Total (*N*)	Univariate analysis		Multivariate analysis	
		Hazard ratio (95% CI)	*P* value	Hazard ratio (95% CI)	*P* value
T stage (T2 & T3 & T4 vs. T1)	370	2.360 (1.745–3.191)	**<0.001**	0.378 (0.049–2.913)	0.350
Pathologic stage (Stage II & Stage III & Stage IV vs. Stage I)	349	2.284 (1.670–3.122)	**<0.001**	4.855 (0.628–37.513)	0.130
Age (>60 vs. ≤60)	373	0.960 (0.718–1.284)	0.783	1.009 (0.702–1.450)	0.962
BMI (>25 vs. ≤25)	336	0.936 (0.689–1.272)	0.673	1.083 (0.747–1.570)	0.673
Histologic grade (G3 & G4 vs. G1 & G2)	368	1.152 (0.853–1.557)	0.355	1.105 (0.759–1.608)	0.603
AFP (ng/ml) (>400 vs. ≤400)	279	1.045 (0.698–1.563)	0.832	0.894 (0.568–1.408)	0.630
SMARCB1 (High vs. Low)	373	1.288 (0.963–1.723)	0.088	1.131 (0.771–1.660)	0.530

Supplementary Table 16. Correlation between TRIM28 expression and clinicopathological characteristics of HCCs.

Clinicopathological variables		TRIM28 expression		P value
		Negative	Positive	
		(*n* = 46)	(*n* = 43)	
Age		52.46 ± 11.49	52.30 ± 10.19	0.947
Sex	Female	4	5	0.734[fisher]
	Male	42	38	
Serum AFP	≤20 ng/ml	19	17	0.865[chisq]
	>20 ng/ml	27	26	
Virus infection (HBV)	HBsAg	10	9	0.926[chisq]
	None	36	34	
Cirrhosis	Absent	5	4	1.000[fisher]
	Present	41	39	
Tumor number	Single	40	39	0.741[fisher]
	Multiple	6	4	
Maximal tumor size	≤5cm	34	28	0.367[chisq]
	>5cm	12	15	
Tumor encapsulation	Absent	21	26	0.162[chisq]
	Present	25	17	
Pathology class	I–II	27	15	0.025[chisq*]
	III	19	28	
AJCC	I	37	26	0.038[chisq*]
	II–III	9	17	
TNM stage	I	37	26	0.038[chisq*]
	II–III	9	17	

Abbreviations: fisher: fisher exact test; chisq: chi-square test; $^{*}p < 0.05$.

Supplementary Table 17. Correlation between H2AX expression and clinicopathological characteristics of HCCs.

Clinicopathological variables		H2AX expression		P value
		Negative	Positive	
		(*n* = 58)	(*n* = 31)	
Age		52.40 ± 11.83	52.35±8.80	0.776
Sex	Female	5	4	0.714[fisher]
	Male	53	27	
Serum AFP	≤20 ng/ml	26	10	0.250[chisq]
	>20 ng/ml	32	21	
Virus infection (HBV)	HBsAg	43	27	0.155[chisq]
	None	15	4	
Cirrhosis	Absent	8	1	0.154[fisher]
	Present	50	30	
Tumor number	Single	54	25	0.090[fisher]
	Multiple	4	6	
Maximal tumor size	≤5cm	38	24	0.245[chisq]
	>5cm	20	7	
Tumor encapsulation	Absent	30	17	0.779[chisq]
	Present	28	14	
Pathology class	I–II	32	10	0.039[chisq*]
	III	26	21	
AJCC	I	46	17	0.016[chisq*]
	II–III	12	14	
TNM stage	I	46	17	0.016[chisq*]
	II–III	12	14	

Abbreviations: fisher: fisher exact test; chisq: chi-square test; $^{*}p < 0.05$.

Supplementary Table 18. Correlation between CDK4 expression and clinicopathological characteristics of HCCs.

Clinicopathological variables		CDK4 expression		P value
		Negative	Positive	
		(*n* = 46)	(*n* = 43)	
Age		53.22±11.56	51.49±10.03	0.454
Sex	Female	4	5	0.734fisher
	Male	42	38	
Serum AFP	≤20 ng/ml	24	12	0.020chisq*
	>20 ng/ml	22	31	
Virus infection (HBV)	HBsAg	37	33	0.671chisq
	None	9	10	
Cirrhosis	Absent	4	5	0.734fisher
	Present	42	38	
Tumor number	Single	43	36	0.188fisher
	Multiple	3	7	
Maximal tumor size	≤5cm	33	29	0.085chisq
	>5cm	13	4	
Tumor encapsulation	Absent	20	27	0.068chisq
	Present	26	16	
Pathology class	I–II	27	15	0.025chisq*
	III	19	28	
AJCC	I	38	15	0.011chisq*
	II–III	8	18	
TNM stage	I	38	25	0.011chisq*
	II–III	8	18	

Abbreviations: fisher: fisher exact test; chisq: chi-square test; $^*p < 0.05$.

Supplementary Table 19. Univariate and multivariate analysis of factors with OS of 83 HCCs.

	Survival					
	Univariate analysis			Multivariate		
	HR	95% CI	*P* value	HR	95% CI	*P* value
Age	0.999	0.964–1.035	0.949			
Sex (female vs. male)	0.000	0.000–Inf	0.997			
Serum AFP (≤20 vs. >20 ng/ml)	1.837	0.767–4.400	0.172			
HBV infection (no vs. yes)	1.083	0.406–2.887	0.874			
Cirrhosis (absent or present)	1.364	0.322–5.786	0.674			
Tumor number (single vs. multiple)	1.251	0.374–4.181	0.716			
Maximal tumor size (≤5 vs. >5 cm)	1.301	0.575–2.946	0.527			
Tumor encapsulation (absent vs. present)	1.473	0.668–3.247	0.337			
TNM stage (II–III vs. I)	2.812	1.279–6.183	0.010*	4.061	0.942–17.514	0.060
Pathology class (III vs. I–II)	2.819	1.177–6.752	0.020*	1.083	0.373–3.125	0.883
AJCC (II–III vs. I)	2.019	0.905–4.506	0.086			
TRIM28 (positive vs. negative)	6.487	2.220–18.953	<0.001	2.310	0.616–8.665	0.124
H2AX (positive vs. negative)	2.492	1.130–5.493	0.024*	1.361	0.497–3.724	0.548
CDK4 (positive vs. negative)	7.313	2.504–21.355	<0.001	3.995	1.119–14.261	0.033*

$^*P < 0.05$.

Supplementary Table 20. Univariate and multivariate analysis of factors with PFS of 83 HCCs.

	Survival					
	Univariate analysis			Multivariate		
	HR	95% CI	*P* value	HR	95% CI	*P* value
Age	1.001	0.966–1.038	0.945			
Sex (female vs. male)	0.337	0.046–2.483	0.286			
Serum AFP (≤20 vs. >20 ng/ml)	1.860	0.819–4.224	0.138			
HBV infection (no vs. yes)	1.027	0.416–2.534	0.954			
Cirrhosis (absent or present)	1.585	0.376–6.681	0.530			
Tumor number (single vs. multiple)	1.090	0.329–3.610	0.888			
Maximal tumor size (≤5 vs. >5 cm)	1.066	0.482–2.356	0.875			
Tumor encapsulation (absent vs. present)	1.476	0.698–3.120	0.309			
TNM stage (II–III vs. I)	2.723	1.292–5.716	0.008**	1.520	0.522–4.424	0.442
Pathology class (III vs. I–II)	2.520	1.110–5.725	0.027*	0.888	0.295–2.672	0.833
AJCC (II–III vs. I)	1.656	0.776–3.537	0.192			
TRIM28 (positive vs. negative)	2.282	1.052–4.951	0.037*	0.702	0.261–1.888	0.483
H2AX (positive vs. negative)	2.360	1.122–4.967	0.024*	1.734	0.708–4.246	0.228
CDK4 (positive vs. negative)	5.545	2.224–13.702	<0.001	4.933	1.290–18.867	0.020*

*$P < 0.05$, **$P < 0.01$.
